# Dynamics in public perceptions and media coverage during an ongoing outbreak of meningococcal W disease in the Netherlands

**DOI:** 10.1186/s12889-022-12920-8

**Published:** 2022-04-01

**Authors:** Marion de Vries, Liesbeth Claassen, Margreet J. M. te Wierik, Danielle R. M. Timmermans, Aura Timen

**Affiliations:** 1 National Institute for Public Health and the Environment (RIVM), Center for Infectious Disease Control / Center for Environmental Safety and Security, Bilthoven, Netherlands; 2 National Institute for Public Health and the Environment (RIVM), Center for Infectious Disease Control, Bilthoven, Netherlands; 3 Department of Public and Occupational Health, Amsterdam UMC, Vrije Universiteit Amsterdam, Amsterdam, Netherlands; 4 VU University Amsterdam, Athena Institute for Research on Innovation and Communication in Health and Life Sciences, Amsterdam, Netherlands

**Keywords:** Meningococcal disease, menACWY, Disease outbreak, Vaccination, Risk perception, Vaccination behavior, Media

## Abstract

**Background:**

From 2015 to 2018, the Netherlands faced an outbreak of invasive meningococcal disease (IMD) caused by serogroup W. To counter the rise in infections, the government introduced a catch-up menACWY vaccination campaign for teenagers in 2018 and 2019. The outbreak situation induced substantial media attention and a run on menACWY vaccines outside the vaccination campaign. This study aimed to gain insights into the dynamics of public perceptions of and responses to the outbreak and the menACWY vaccination, and into the media coverage about the outbreak.

**Methods:**

Three repeated surveys (*N* = 1110) between 2017 and 2019 were sent to parents of teenagers invited for a menACWY catch-up vaccination, other parents, and individuals with no under-age children. These surveys assessed IMD risk perceptions, attitudes towards the menACWY vaccination, trust in involved institutions, and willingness to vaccinate with the menACWY vaccine. Changes in the public perceptions and responses were studied with linear multilevel regression analyses. In addition, 103 national newspaper articles from the period 2017–2019 were thematically coded with themes about IMD and the menACWY vaccination.

**Results:**

The survey results showed clear increases in perceived IMD severity, positive attitude towards the menACWY vaccination, and willingness to vaccinate over time. Perceived IMD vulnerability remained low across all three waves, and trust in involved institutions increased slightly. Differences between the survey groups were limited. The newspaper articles discussed the rise in infections extensively, the disease symptoms, and the possible fatal outcome of IMD. In addition, while many articles discussed the menACWY vaccine shortage, few discussed the safety or effectiveness of the vaccine.

**Conclusion:**

The real-time insights into the interrelated dynamics of public perceptions, responses, and media coverage provide an integrated portrait of the social developments during this outbreak. The focus on IMD severity and the absence of doubt in the public discussion about vaccine safety may have played an important role in the societal response to this outbreak and the recommended vaccine.

**Supplementary Information:**

The online version contains supplementary material available at 10.1186/s12889-022-12920-8.

## Background

Between 2015 and 2018, the number of invasive meningococcal disease (IMD) cases caused by serogroup W increased rapidly in the Netherlands. The number of patients with IMD W increased from an annual average of four cases before 2015 to 103 cases in 2018 (see Supplementary file 1 for historical data of the IMD burden from serogroup W and other serogroups in the Netherlands). IMD W infections and fatalities were seen in all age groups, but young children, adolescents, and elderly people were most affected [1].

**Fig. 1:**
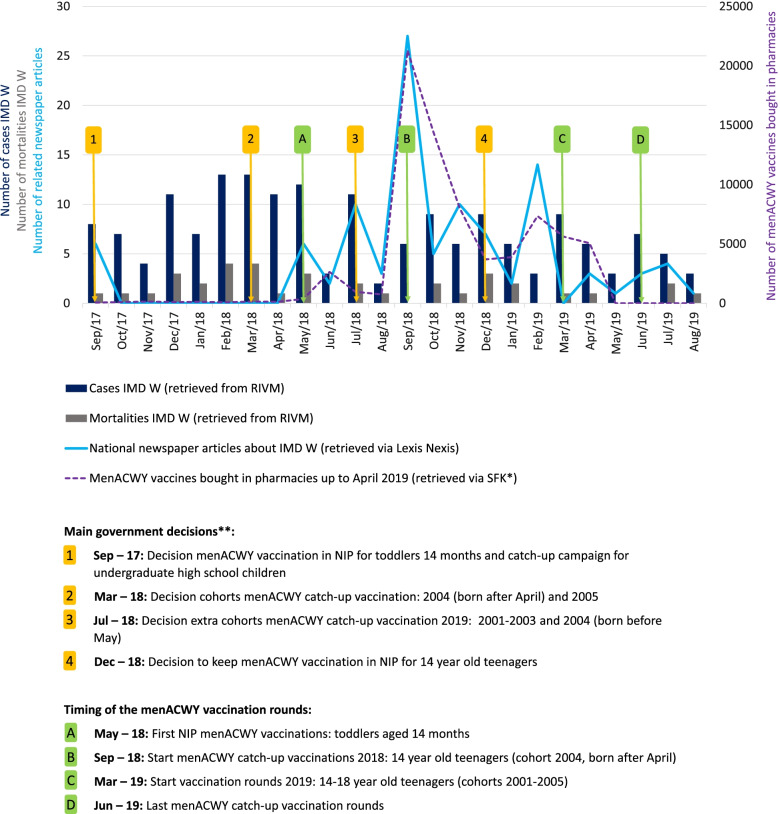
A timeline of the IMD outbreak situation between September 2017 and September 2019. *Foundation Pharmaceutical Key Figures. These figures are a proxy for the number of vaccines administered outside the National Immunization Program and catch-up campaign, for example by general practitioners at the request of their patients. These figures exclude the vaccines administered by the public health services (GGD), who were in charge of administering the vaccines in the National Immunization Program and the catch-up campaign. These figures have been published elsewhere [2, 3]. ** Due to limited menACWY vaccine availability, the government had to adapt its policy regarding the menACWY catch-up vaccination campaign over the course of 2017–2019. In September 2017 (1 in figure), the Dutch government announced plans to implement a catch-up menACWY vaccination campaign for junior-high-school children (usually aged 12–14 years old). In March 2018 (2), the government communicated that due to the limited vaccine availability only children born between May and December 2004 (aged ~ 14 years old) would be invited for catch-up vaccination in 2018 and that children born in 2005 would be invited for vaccination in 2019. In July 2018 (3), the target group for the catch-up vaccination in 2019 was extended to all children born between 2001 and 2005 (all aged 14–18 years old, excluding those who would receive the vaccination in 2018) as more vaccines had become available. In December 2018 (4), it was decided to offer the menACWY vaccine in subsequent years to all 14 year old teenagers via the National Immunization Program

A menACWY conjugate vaccine was introduced in the National Immunization Program (NIP) for children aged 14 months starting in 2018 to counter the rapid rise in infections. In addition, teenagers aged 14–18 years were invited for a catch-up menACWY vaccination campaign in 2018 and 2019 [1]. The catch-up vaccination campaign for teenagers was complicated due to menACWY vaccine shortages that resulted in changing policies regarding the age groups invited for the vaccination over the course of 2017–2019 (see Fig. 1). The catch-up vaccination campaign was accompanied with an extensive awareness and information campaign for teenagers and their parents, in which public health authorities stressed the importance of getting vaccinated in order to protect oneself and one’s friends. The campaign with the slogan ‘Do not share this with your friends. Get that shot against meningococcal disease.’ was delivered through a variety of channels and with a variety of tools to underpin the main message.

Despite the shortages in vaccine supplies, the vaccination campaign succeeded in reaching most of the teenagers invited (vaccination coverage: 84%). In parallel, a rapid increase in the uptake of menACWY vaccines outside the NIP and the catch-up campaign was seen (see Fig. 1) [2]. In the Netherlands, residents can ask their general practitioner for an (out-of-pocket) vaccination if they are not eligible for a free vaccine in a vaccination campaign or the NIP. Insights into both the responses of those targeted for vaccination and those not targeted for vaccination are important in the evaluation of this vaccination campaign. To understand these public responses, we need insights into public perceptions of the disease risk, perceptions of the vaccination, and trust in the involved institutions over the outbreak period. These factors are determinants of vaccination intentions [4, 5] and important indicators of public sentiments. As people’s perceptions of the disease threat, their perceptions of the vaccination, and their trust in institutions are subject to change as the outbreak unfolds [6–11], it is important to study these public perceptions and responses over time. Earlier studies examined perceptions of IMD [12–19] and perceptions of the menACWY vaccination [16, 17, 19, 20]*,* but all these studies were cross-sectional.

To better understand (changes in) public perceptions and responses during an outbreak situation, insight into the media coverage during the outbreak is valuable. While governments and public health institutes are often the first sources of new information about an outbreak situation, the (traditional) media are probably the most important disseminator of this information to the public [21, 22]. Journalists adopt new information from authorities and translate this information into newsworthy items for the public. In this process, the media can influence what people know and believe [23–29]. At the same time, public sentiments might also influence media coverage, as media outlets are likely to adapt their products to the interests and sentiments of the public [30].

The aim of this study was to provide an integrated portrait of two important interrelated social dynamics during an emerging outbreak of IMD in the Netherlands, namely public perceptions and responses, and media coverage. We studied the dynamics of public perceptions of the risks posed by IMD, perceptions of the menACWY vaccination, trust in involved institutions, and willingness to receive the menACWY vaccination between September 2017 and September 2019. These factors were studied among parents of children targeted for a menACWY vaccination, parents of children who were not targeted for a menACWY vaccination, and other adults. To be able to put the changes in perceptions and responses into context, we additionally analyzed the national newspaper coverage about the risks posed by IMD, the menACWY vaccination, and the involved institutions in this same period.

## Methods

This paper reports on a study that focuses on 2 units of analysis. We studied public perceptions and responses by means of a quantitative analysis of the data from three repeated surveys, and we studied media coverage by means of a qualitative content analysis of national newspaper articles.

### The repeated surveys: study population and procedure

Three repeated online surveys were collected via an online survey panel (Flycatcher Internet Research, ISO 26362). This is an opt-in survey panel, meaning that people can join the panel population without invitation. Panel members save points with survey participation which can be exchanged for gift vouchers or charity donations. Small incentives for participation limit non-response bias and do generally not strongly affect the sample composition [31]. The surveys were sent to a selection of the panel population (which consists of approximately 10,000 active members), namely to 784 (first survey) individuals (aged 18+) who were preselected to represent the general Dutch population (aged 18+) based on their sex, age, education, income, and region of residence, and 842 (first survey) parents of children under the age of 18 who were preselected to represent the population in their age group (25–55 years) based on their sex, education, and region of residence. The selection of the study participants was purposively rather than randomized, aiming at a respondent population representative of the population at large based on demographic characteristics. The first survey (S1) was sent in December 2017, approximately 3 months after the decision to have a menACWY catch-up campaign (see Fig. 1). The second survey (S2; sent to all participants from the first survey) was collected in September 2018, approximately 2 weeks before the first menACWY catch-up vaccinations. The third survey (S3; sent to all participants from the second survey) was collected in July 2019, shortly after the final catch-up vaccination rounds were finished.

Participation in the survey was voluntary (panel members save credits for vouchers), and the panel members were informed about the general purpose of the study prior to participation. Written consent for data sharing was given by each panel member prior to their registration to the survey panel. The Clinical Expertise Center RIVM has reviewed the study protocol and concluded that this research was not subject to the Dutch law for medical research involving human subjects [32]. Our study was, therefore, exempted from seeking further approval from an Ethical Research Committee.

### The repeated surveys: measurements and design

The survey questions addressed perceptions of the risk posed by IMD (W), perceptions of the menACWY vaccination, trust in involved institutions (the RIVM, the government, and pharmaceutical companies), and willingness or intention to receive the menACWY vaccination. An overview of the measurements discussed in this paper is shown in Table 1. In addition, all respondents were asked if they had children under the age of 18 years old, and if yes, what the age of their children was.

**Table 1:** Survey measures

*Variable*	*Survey question and items*^a^	*Answer categories*	*Cronbach’s Alpha*^b^
MenACWY vaccination intention – child	Do you want your youngest child to be vaccinated against meningococcal disease type A, C, W, and Y?	*0 certainly not – 6 certainly yes*	–
MenACWY vaccination intention – self	Do you want to be vaccinated against meningococcal disease type A, C, W, and Y?	*0 certainly not – 6 certainly yes*	–
Perceived probability IMD – child	- Do you think that your youngest child may get sick due to meningococci in the following 12 months? In your opinion, how likely is this?	*0 very unlikely – 6 very likely*	0.9
	- Do you think that your youngest child may get sick due to meningococci in his/her life? In your opinion, how likely is this?	*0 very unlikely – 6 very likely*	
Perceived probability IMD – self	- Do you think that you may get sick due to meningococci in the following 12 months? In your opinion, how likely is this?	*0 very unlikely – 6 very likely*	0.8
	- Do you think that you may get sick due to meningococci in your life? In your opinion, how likely is this?	*0 very unlikely – 6 very likely*	
Perceived severity IMD – child	How would it be for you if your youngest child got sick due to meningococci?	*0 not at all severe – 6 very severe*	–
Perceived severity IMD – self	How would it be for you if you got sick due to meningococci?	*0 not at all severe – 6 very severe*	–
Attitude menACWY vaccination (negative – positive)	[…] What do you think about the decision regarding the vaccination against meningococci type A, C, W, and Y? I think it is …		0.9
	- 0 unnecessary – 6 necessary		
	- 0 acceptable – 6 unacceptable^c^		
	- 0 safe – 6 dangerous^c^		
	- 0 poor – 6 good		
	- 0 Not self-evident – 6 self-evident		
Trust in government	[…] What do you think about our government when it comes to infectious diseases and vaccinations? […]	*0 completely disagree, 1 disagree 2 do not agree nor disagree, 3 agree, 4 completely agree*	0.9
	- The government has sufficient knowledge and skills with regard to infectious diseases and vaccinations.		
	- The government communicates openly about infectious diseases and vaccinations.		
	- The government puts the health of citizens above economic interests when it comes to vaccinations		
Trust in RIVM	[...] What do you think about the RIVM when it comes to infectious diseases and vaccinations? […]	*0 completely disagree, 1 disagree 2 do not agree nor disagree, 3 agree, 4 completely agree*	0.9
	- The RIVM has sufficient knowledge and skills with regard to infectious diseases and vaccinations.		
	- The RIVM communicates openly about infectious diseases and vaccinations.		
	- The RIVM puts the health of citizens above economic interests when it comes to vaccinations		
Trust in pharmaceutical companies	[...]What do you think about pharmaceutical companies when it comes to infectious diseases and vaccinations? […]	*0 completely disagree, 1 disagree 2 do not agree nor disagree, 3 agree, 4 completely agree*	0.7
	- Pharmaceutical companies have sufficient knowledge and skills with regard to infectious diseases and vaccinations.		
	- Pharmaceutical companies communicate openly about infectious diseases and vaccinations.		
	- Pharmaceutical companies put the health of citizens above economic interests when it comes to vaccinations		

^a^See Supplementary file 2 for the exact wording of questions and items per survey (small adaptations were made in follow-up surveys to adopt to changing context and insights)

^b^Indicating internal scale consistency with a value between 0 (minimum) and 1 (maximum)

^c^For these items, the scale was inverted prior to constructing the scale

Perceptions of IMD were operationalized as the perceived probability of contracting IMD (two questions) and the perceived severity of contracting IMD (one question). Perceptions of the menACWY vaccination were studied by asking the respondents about their attitude with regard to the menACWY vaccination (policy) at that point in time (one question, four items). Prior to this question in each survey wave (S1-S3), respondents read a short text with information about the vaccination policy at that moment (see the full surveys in Supplementary file 2). These texts were added to the questionnaires because the target groups of the menACWY vaccination campaign changed during the outbreak period (see Fig. 1). Trust in institutions was measured with three questions (one for each institution: the government, the RIVM, and pharmaceutical companies) with three items each (assessing the perceived capability, openness/honesty, and care/concern) [33]. Willingness to receive the menACWY vaccination was measured with a single question.

Each parent in the sample answered the questions regarding perceived IMD probability, perceived IMD severity, and willingness to vaccinate both regarding themselves (e.g., “do you want to get vaccinated”) as regarding their youngest child (“do you want your youngest child to get vaccinated?”).^1^ Individuals with no children under the age of 18 years received these questions only regarding themselves.

### The repeated surveys: statistical analysis

The total respondent population was split into three non-overlapping groups for the analyses: Parents whose youngest child is a teenager targeted for a menACWY vaccination (from now on referred to as parents (T), T for ‘teenagers’), other parents whose youngest child (aged < 18 years) was not targeted for a menACWY vaccination (referred to as parents (O), o for ‘other children’), and individuals with no children under the age of 18 (referred to as individuals (NC), NC for ‘no children’). Parents whose youngest child was aged < 2 years, and thus potentially eligible for the menACWY vaccination in the NIP for toddlers aged 14 months, were excluded from the sample due to the limited number of respondents in this group (*N* = 41).

Based on principal component analyses (PCA) and reliability analyses, constructs were developed for concepts including multiple questions and items (Cronbach alphas are shown in Table 1. Cronbach alpha’s indicate internal scale consistency with a value between 0 (minimum) and 1 (maximum). Values between 0 .7 and 0.9 are generally considered acceptable for a reliable scale [34]). Descriptive statistics (means and standard deviations) were produced for all variables in Table 1 in each group of respondents. In these descriptive analyses, only respondents who participated in each wave (S1-S3) were included. Differences in demographic characteristics (age, sex, education level) and subgroups (parents (T), parents (O), and individuals (NC)) at S1 were studied between respondents who participated in all three surveys (S1-S3) and respondents who participated only in S1 or in S1 and S2. This was done with independent t-tests (for variables with a ratio scale) and chi^2^ tests (for categorical variables). Finally, we performed linear multilevel analyses to study changes in all variables in Table 1 between the three waves (main effect time), and to study differences between groups, overall (main effect group) and in changes over time (interaction effect group X time). In these analyses, we compared individuals (NC) with all parents (T and O) to explore differences in perceptions and responses based on having under-age children or not. We consequently compared parents (T) with parents (O), to see whether perceptions and responses in parents of children who were targeted for a vaccination differed from those in parents of children who were not targeted for vaccination. All multilevel analyses were controlled for the respondents’ age, sex, and education level.

### Newspaper articles: data retrieval and selection

Newspaper articles about meningococcal (W) disease and/or the menACWY vaccination from nine Dutch national newspapers^2^ were retrieved via the Lexis Nexis search operator [35]. The search term used was meningococ! (in Dutch: *meningokok*!). With this search term, all articles with a word that started with meningococ were recovered (e.g., meningococci or meningococcal disease). The search resulted in 148 articles in the period from September 2017 to September 2019. Articles that reported solely about another meningococcal serogroup (e.g. meningococcal B, 8 articles) or that contained less than a paragraph on the topic of meningococcal (W) disease and/or the menACWY vaccination (37 articles) were excluded from the analysis. 103 articles were found eligible for the analysis.

### Newspaper articles: content analysis

An explorative inductive analysis of the newspaper articles was done by an intern and the first author (MdV) from which various recurrent themes were identified about meningococcal (W) disease, the menACWY vaccination, and the government’s policy regarding the menACWY vaccination. In the final analysis, each article was coded per paragraph with the presence or absence of each of the themes in the analysis program Atlas.ti by the first author (MdV). In addition to these themes, we coded the presence/absence of references to important institutions involved in menACWY vaccination policy and implementation, namely the National Institute for Public Health and the Environment (RIVM), the national government, and pharmaceutical companies.

Author LC coded a subset of the dataset (15%) after the final analysis. Consequently, interrater reliability was assessed per code (themes and references to institutions) with the Kappa coefficient (an estimate of agreement between coders, controlled for chance) [36].

## Results

### The repeated surveys

The response rate of the total survey population was 68% (*N* = 1110/1626) for wave 1, 71% at wave 2 (*N* = 784/1110), and 71% at wave 3 (*N* = 558/784). Among the respondents for wave 1, 213 were categorized as parents (T), 392 as parents (O), and 464 as individuals (NC). A description of the survey population is shown in Table 2. Respondents who participated in all survey waves (S1-S3) were (at S1) slightly older than those who participated only in S1 or in S1 and S2, and the drop-out was slightly higher among those with lower education and among parents (O) (see Supplementary file 3).

**Table 2:** Description of survey respondent groups in frequencies (N), percentages (%), means (M), and standard deviations (SD)

*Respondent group*^a^	*Parents (T)*			*Parents (O)*			*Individuals (NC)*		
*Survey wave*^b^	*S1*	*S2*	*S3*	*S1*	*S2*	*S3*	*S1*	*S2*	*S3*
*Female – N (%)*	116 (54.5)	80 (53.7)	57 (51.4)	214 (54.6)	148 (57.4)	101 (58.0)	216 (46.6)	165 (47.4)	118 (45.9)
*Age in years – M (SD)*	47.6 (5.7)	47.8 (5.9)	48.9 (6.2)	40.9 (7.0)	41.2 (7.2)	42.3 (7.3)	53.0 (16.9)	54.0 (16.5)	56.6 (16.1)
*Education – N (%)* *- Low*	101 (47.4)	65 (43.6)	42 (37.8)	118 (30.1)	66 (25.6)	40 (23.0)	127 (27.4)	96 (27.6)	63 (24.5)
*- Intermediate*	74 (34.7)	52 (34.9)	44 (39.6)	130 (33.2)	94 (36.4)	71 (40.8)	209 (45.0)	162 (46.6)	129 (50.2
*- High*	38 (17.8)	32 (21.5)	25 (22.5)	144 (36.7)	98 (38.0)	63 (36.2)	128 (27.6)	90 (25.9)	65 (25.3)
*Already menACWY vaccinated – N (%)*	12 (5.6)	6 (4.0)	9 (8.1)	16 (4.1)	15 (5.8)	10 (5.7)	13 (2.8)	13 (3.7)	21 (8.2)
*Youngest child already menACWY vaccinated – N (%)*	16 (7.5)	21 (14.1)	98 (88.3)	28 (7.1)	37 (14.3)	38 (21.8)	–	–	–
*Total – N*	213	149	111	392	258	174	464	348	257

^a^Parents of teenagers invited for the menACWY catch-up vaccination campaign (parents (T)), parents of children under the age of 18 who were not invited for a menACWY vaccination (parents (O)), and individuals with no children under the age of 18 (individuals (NC))

^b^First survey (S1), second survey (S2), and third survey (S3)

#### Dynamics in IMD risk perceptions, attitude towards the menACWY vaccination and trust in institutions

**Fig. 2:**
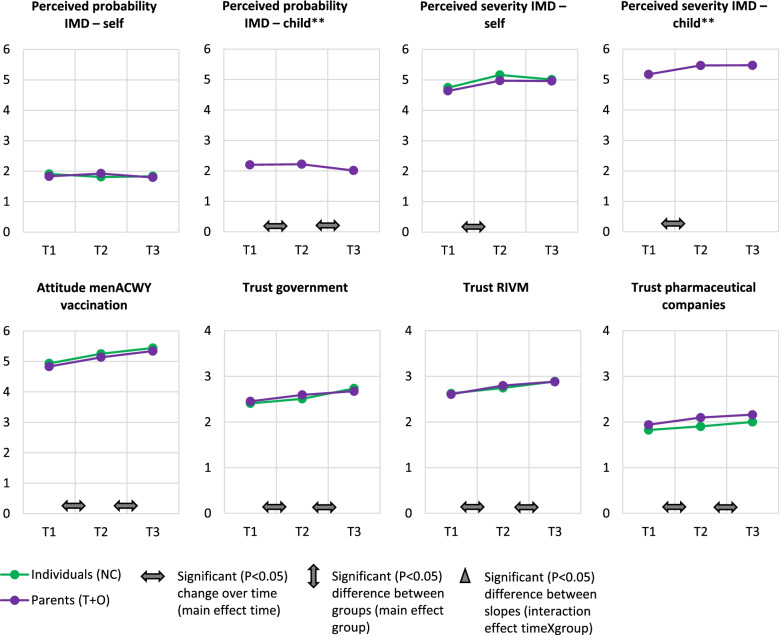
Mean values of risk perceptions, attitude towards the menACWY vaccination policy and trust in institutions at S1-S3, and the results from the multilevel analyses* in parents (of teenagers (T) and other children (O)) and individuals with no under age children (NC). * Tables with the descriptive analyses (means and standard deviations) and the results from the multilevel analyses are shown in Supplementary file 4. Multilevel results shown in this figure are the significante (*p* < 0.05) changes in the variables between the consequent waves. There were no significant differences between the groups observed, overall, or in slopes. ** Only assessed among parents

Figure 2 shows perceptions and responses among parents (T and O together) and individuals (NC). Perceived personal vulnerability to IMD in both parents (T and O) and individuals (NC) was low and stable over the three waves. Perceived personal severity of contracting IMD was considerably higher and increased between S1 and S2 (β = 0.4 (95% confidence interval (CI) = 0.3, 0.5)).

Parents’ (T + O) perceived vulnerability regarding their child contracting IMD increased slightly but significantly (*p* < 0.05), between S1 and S2 (β = 0.1 (0.0, 0.3)), and decreased between S2 and S3 (β = − 0.4 (− 0.5, − 0.2)). Parents’ perceived severity of their child contracting IMD also increased between S1 and S2 (β = 0.3 (0.2, 0.4)).

Both parents and individuals (NC) had a fairly positive attitude towards the menACWY vaccination (see Fig. 2), and this positive attitude increased between S1 and S2 (β = 0.3 (0.2, 0.4)) and S2 and S3 (β = 0.2 (0.1, 0.3)). The mean values for trust in the government and the RIVM were higher than those for trust in pharmaceutical companies. Trust in all three institutions increased slightly but significantly (*p* < 0.05) over the three waves with coefficients between 0.1 and 0.2 (see Supplementary file 4). No significant differences were found in perceptions nor in trust between parents and individuals (NC).

**Fig. 3:**
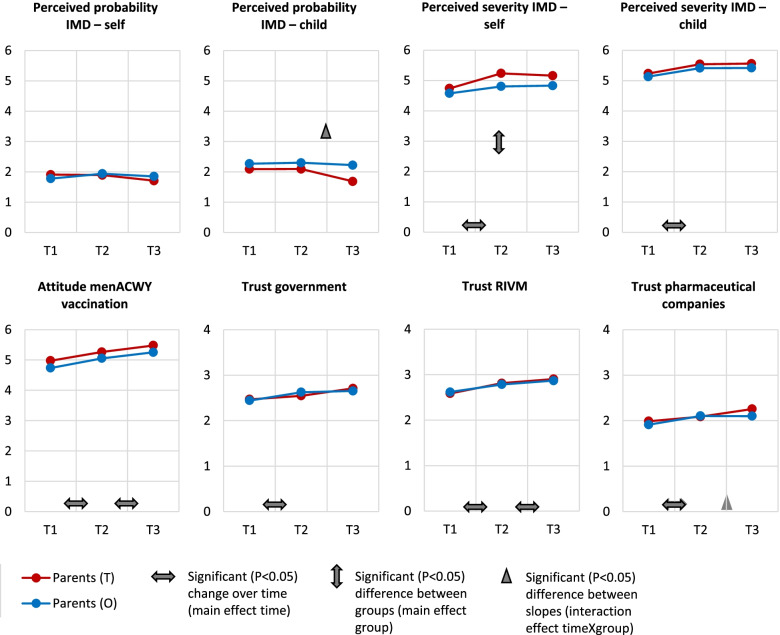
Mean values of risk perceptions, attitude towards the menACWY vaccination policy and trust in institutions at S1-S3, and the results from the multilevel analyses* in parents of teenagers (T) and parents of other under-age children (O). * Tables with the descriptive analyses (means and standard deviations) and the results from the multilevel analyses are shown in Supplementary file 4. Multilevel results shown in this figure are the significant (*p* < 0.05) changes in the variables between the consecutive waves, the significant (*p* < 0.05) overall differences between groups, and the significant (*p* < 0.05) differences in slopes between groups

Figure 3 shows perceptions and responses among parents (T) and parents (O) separately. Only two significant differences were found between parents (T) and parents (O). Between S2 and S3, perceived vulnerability regarding their child contracting IMD declined stronger in parents (T) compared to parents (O) (β = − 0.6, 95% confidence interval (CI) = − 0.9, − 0.3). In addition, parents (T) perceived their personal severity if contracting the disease as higher than parents (O) did (β = 0.4, 95% CI = 0.1, 0.6).

#### Dynamics in willingness to vaccinate

**Fig. 4:**
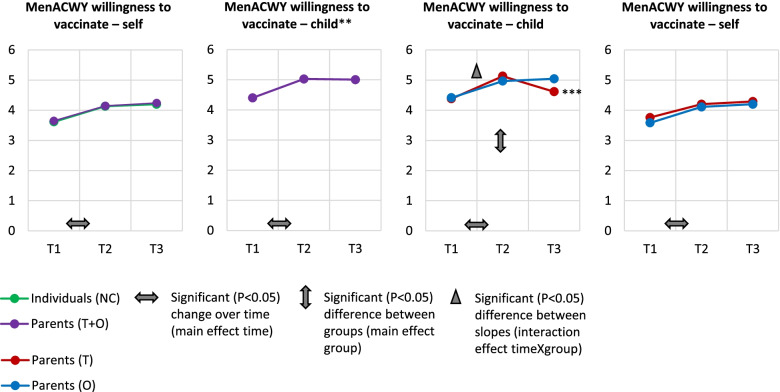
Mean values of willingness to vaccinate (both with regard to one’s child as to oneself) and results from the multilevel analyses*, in parents (of teenagers (T) and other children (O)) and individuals with no under-age children (NC) on the one hand, and in parents (T) and parents (O) separately. * Tables with the descriptive analyses (means and standard deviations) and the results from the multilevel analyses are shown in Supplementary file 4. Multilevel results shown in this figure are the significant (*p* < 0.05) changes in willingness to vaccinate between the consecutive waves and the significant overall differences between groups. There were no significant (*p* < 0.05) differences in slopes between groups. ** Only assessed among parents. *** During wave 3, only 13 parents (T) answered the question of vaccination intention for their child because the majority of the respondents indicated that their child had already received the menACWY vaccination

Figure 4 shows the willingness to vaccinate with the menACWY vaccination among the respondents. Willingness to have themselves vaccinated with the menACWY vaccination increased between S1 and S2 (β = 0.5, 95% CI = 0.3, 0.7) among parents and individuals (NC). Parents and Individuals (NC) did not differ significantly in their willingness to have themselves vaccinated.

Parent’s (T + O) willingness to vaccinate their child also increased between S1 and S2 (β = 0.5, 95% CI = 0.3, 0.6). The willingness to vaccinate their child was slightly, though significantly (*p* < 0.05), higher among parents (T) than among parents (O) (β = 0.3, 0.0, 0.7) and increased more strongly in parents (T) between S1 to S2 (β = 0.4, 95% CI = 0.1, 0.6).

### The content analysis of newspaper articles

Between September 2017 and September 2019, 103 national newspaper articles were published about meningococcal W disease and/or the menACWY vaccination (see Fig. 1). Prior to the first survey wave (S1), 6 articles were published, 41 articles between the first (S1) and second survey (S2), and 55 between the second (S2) and third survey (S3). One article was published between the third survey (S3) and September 2019 (see Supplementary file 5 for details).

The main themes identified in the newspaper articles were *IMD vulnerability*, *IMD severity,* and *menACWY vaccination*. An overview of the identified (sub-)themes and the references to institutions (government, RIVM, and pharmaceutical companies) is shown in Table 3. The interrater reliability was sufficient (mean Kappa coefficient: 0.85).

**Table 3:** Recurrent themes in the newspaper articles

Theme	Sub-themes	Description	Exemplifying quote
1. Probability IMD	a. Statistics	Information about the number of morbidity and/or mortality cases in the Netherlands	*This year, 65 people have become seriously ill with type W meningococci. Of these, 15 died, four more than in all of 2017.*^a^
	b. Rise in infections	A verbal description of the rise in meningococcal (W) disease	*Since 2015, more adolescents have fallen ill with meningococci. In addition, more people die of meningococcal disease every year.*^b^
	c. Individual risk	A verbal description of the probability of developing meningococcal (W) disease for an individual	*“Despite the rise, the probability of becoming infected is very small, but to prevent worse, we have to intervene,” says [name Secretary of State].*^c^
	d. Relative risk	A description of the risk of infection for one group compared with another group or the general population	*“Meningococcal disease occurs in all age groups, but especially in young children, teens, and the elderly.*^d^
2. Severity IMD	a. Disease information	Information about symptoms and/or disease progression and/or long-term consequences of IMD (W)	*The meningococci can spread at lightning speed, cause blood poisoning, and affect the meninges, which can cause a person to die within 24 h.*^e^
	b. Fatality	Reference to the possible fatal consequences of IMD (W)	*What may be perceived as a serious case of the flu can lead to a life-threatening infection in a short period, in which the patient has to be treated in the intensive care unit of the hospital.*^f^
	c. Individual cases	References to/stories about individuals who contracted IMD (W) disease	*After an agony of almost three weeks, his parents turned off the ventilation. [name], a 17-year-old, until then always healthy, boy, is dead.*^g^
3. MenACWY vaccination	a. Vaccine safety/ effectiveness	Information about the effectiveness, duration of protection and/or safety of the menACWY vaccine	*This vaccine is very likely to work well. It has few side effects and it is expected to also provide group protection.*^h^
	b. Vaccine shortage	Information about the menACWY vaccine shortage and the increase in demand for the menACWY vaccine outside the vaccination campaign	*Those who go to the doctor now to get vaccinated against meningococcal type W, are out of luck. “Because there are not enough vaccines, we are choosing to give it to the 14-year-old children,” says [name RIVM employee].*^i^
	c. Questioning policy	Texts that question the policy regarding the timing of the implementation and/or the choice of target groups for the menACWY vaccination	*,I think it is strange that us citizens have not been informed before. This disease is a silent killer; even if you survive it you could be disabled. The government could have acted earlier […]*^j^
1.**Reference to institutions**	**Institution**	**Description**	**Exemplifying quote**
	a. Government	References to the government in relation to the IMD/menACWY situation	*State Secretary [name] (Public Health) starts the large-scale additional vaccination campaign because the insidious infectious disease has been claiming more and more victims in recent years.*^k^
	b. RIVM	References to the RIVM in relation to the IMD/menACWY situation	*The National Institute for Public Health and the Environment (RIVM) launched a new vaccination campaign against meningococcal disease targeting young people on Monday afternoon.*^l^
	c. Pharmaceutical companies	References to a pharmaceutical company or pharmaceutical companies in general in relation to the IMD/menACWY situation	*In an email from September 2016, seen by [name newspaper], drug manufacturer [name] points out to the National Institute for Public Health and the Environment (RIVM) that the number of cases due to meningococcal type W has risen sharply.*^m^

^a^Nieuwenhuis, M. (2018, July 19). Meningococci increasingly deadly, especially among teenagers (In Dutch: Meningokokken steeds dodelijker, vooral bij tieners). Algemeen Dagblad, p.7

^b^De Jong, M. (2019, April 19) Inoculation factory against meningococci (In Dutch: Prikfabriek tegen meningokokken). De Telegraaf, p.15

^c^Nieuwenhuis, M. (2018, July 18). Large vaccination campaign against meningococci (In Dutch: Grote inentingsactie tegen meningokokken). Algemeen Dagblad, p.6

^d^Speksnijder, C. (2018, May 31). From Chile via England: Meningococcus is back (In Dutch: V a n u i t C h i l i v i a E n g e l a n d : d e m e n i n g o k o k i s t e r u g ). De Volkskrant, p.12

^e^Nieuwenhuis, M. (2019, July 10). Deadly bacteria is shedding (In Dutch: Dodelijke bacterie legt het af). Algemeen Dagblad, p.7.

^f^Voormolen, S. (2018, September 11). In a hurry with vaccination against meningococci (In Dutch: Haast met vaccinatie tegen meningokokken). NRC Handelsblad, p.8

^g^Ten Broeke, A. (2018, November 2). Vaccinating in times of capitalism (In Dutch: Vaccineren in tijden van kapitalisme). De Volkskrant, p.25

^h^Nieuwenhuis, M. (2018, December 19). “Inoculation against type W useful for all Dutch people” (In Dutch: “Prik tegen type W nuttig voor álle Nederlanders”). Algemeen Dagblad, p.5

^i^Nieuwenhuis, M. (2018, July 1). Shortage of vaccines against deadly bacteria (In Dutch: Tekort vaccins tegen dodelijke bacterie). Algemeen Dagblad, p.9

^j^Van der Kaaden, A. (2018, December 31). “Our youngest daughter recently asked me: Mom will you ever be happy again?” (In Dutch: “Onze jongste dochter vroeg me laatst: mam word je weer gelukkig?”). NRC Handelsblad, p.1

^k^Lengton, I. (2018, July 17). New inoculation offensive (in Dutch: Nieuw prikoffensief ). De Telegraaf, p.7

^l^Unknown author. (2018, September 10). RIVM starts campaign against communicable disease meningococcus (In Dutch: RIVM start campagne tegen infectieziekte meningokok). Reformatorisch Dagblad, p.12

^m^Efting, M. & Misérus, M. (2018, September 14). Government knew about meningococcus danger in 2016 (In Dutch: Rijk wist in 2016 al van meningokok-gevaar). De Volkskrant, p.1

#### Newspaper coverage <S1 (1 august 2017–12 December 2017)

All six articles in the period before the first survey (<S1) were published on 26 September 2017 and reported about the government’s decision to introduce the menACWY vaccination into the NIP for toddlers aged 14 months and in a catch-up campaign for junior-high-school children (see Fig. 1). With regard to the theme of IMD probability, all articles reported on the *rise in infections,* two out of six articles mentioned IMD *statistics* in the Netherlands, and three discussed *relative risks*. The theme of IMD severity was seen in five articles that provided *disease information* and in one article that mentioned possible *fatality* due to IMD. Regarding the theme of menACWY vaccination, two articles mentioned *menACWY vaccine shortage*. The *government* was the only institution mentioned in all six articles.

#### Newspaper coverage S1-S2 (13 December 2017–20 September 2018)

**Fig. 5:**
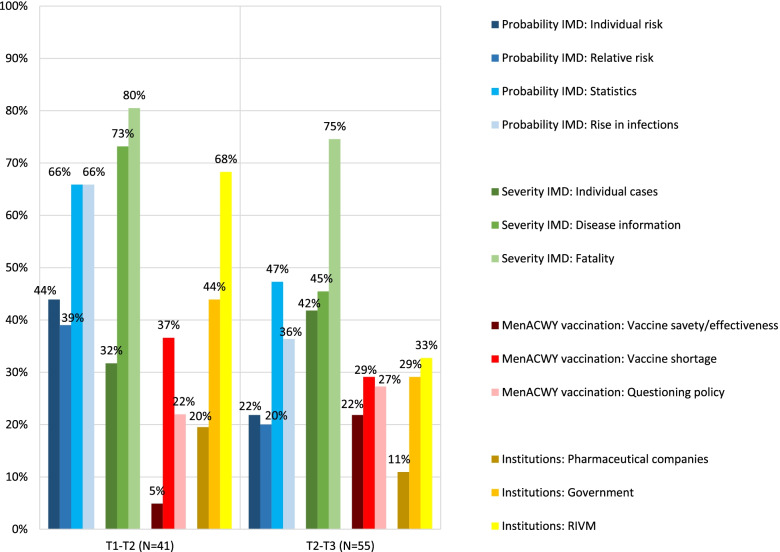
Percentages of newspaper articles with a theme and/or reference to an institution in S1-S2 and S2-S3*. * The results from period <S1 are not shown here because of the limited number of articles (*N* = 6)

The 41 articles in the period between the first and second surveys (S1-S2) were published in May (7), June (2), July (11), August (4), and September (21) 2018. In this period, a majority of the articles discussed IMD probability in terms of morbidity/mortality *statistics* and/or the *rise in infections* (see Fig. 5). More than a third of the articles mentioned the probability of falling ill due to IMD for the individual (*individual risk*) and/or *relative risks* (the risk of one group compared with another). Most of the articles that did mention the *individual risk* stated that the risk of being infected was small. Most articles that discussed *relative risks* emphasized that young children, adolescents and/or the elderly were at increased risk of an IMD infection. A majority of the articles in S1-S2 discussed IMD severity with *disease information* and/or mentioning possible *fatality* due to IMD. More than a third of the articles reported on *individual cases* of IMD. Most of these articles discussed adolescents that had suffered or died from IMD; some briefly mentioned the death of an adolescent; others provided detailed narratives.

Regarding the menACWY vaccination, many articles reported on the decision to invite more teenagers (all aged 14–18 years) in 2019 for the menACWY vaccination (mainly articles from July 2018), and many articles reported about the start of the menACWY campaign for 14-year-olds (mainly articles from September 2018, see also Fig. 1 for the main decisions in vaccination policy and the timing of the vaccination rounds). In addition, in the period S1-S2, more than a third of the articles mentioned the *vaccine shortage,* and in about a fifth of the articles vaccine policy was questioned or criticized (*questioning vaccine policy,* see Fig. 5). Only 5 % of the articles mentioned the vaccine’s *safety or effectiveness*. Of the three institutions, the RIVM was mentioned most often (68%, often in relation to the start of the menACWY vaccination campaign), followed by the government (44%, often in relation to the decision to invite more teenagers for the menACWY vaccination), and the pharmaceutical companies (20%, often in relation the vaccine shortage and questioning the vaccine policy).

#### Newspaper coverage S2-S3 (21 September 2018–14 July 2019)

Of the 55 articles in the period S2-S3, most were published from September–December 2018 [30] and in February 2019 (14, see Fig. 1). Compared with the period S1-S2, we observed lower percentages of articles that were coded with one of the IMD probability sub-themes (see Fig. 5). With regard to IMD severity, a higher percentage of articles discussed *individual cases* with IMD, but considerably fewer articles gave *disease information.* A majority of the articles still mentioned the possible *fatality* due to IMD, although the percentage was slightly smaller compared with S1-S2.

With regard to the menACWY vaccination, several articles discussed the vaccination rounds in 2018 and 2019, the vaccination uptake among teenagers, and the decision to keep the menACWY vaccination in the NIP for 14-year-olds (see main policy decisions in Fig. 1). The percentage of articles that discussed the vaccine’s *safety or effectiveness* was higher compared with S1-S2. Quotes addressing the vaccine safety or effectiveness were mostly framed factually or positively (e.g. “specialists assume that there is protection for up to five years”^3^ and “This vaccine is very likely to work well. It has few side effects and it is expected to also provide group protection”^4^). The percentages of articles coded with *questioning vaccine policy* and *vaccine shortage* were slightly lower in this period, and all three institutions were mentioned considerably less often than in S1-S2.

## Discussion

Our findings regarding public perceptions, responses, and newspaper coverage together provide an integrated portrait of the social developments during the IMD outbreak. In general, the public seemed well aware of the threat posed by the rising meningococcal W infections, was positive about the menACWY vaccination, trusted the involved institutions, and was willing to vaccinate against IMD. The newspapers provided elaborate information on the rise in infections and severity of the disease symptoms. And when addressing the menACWY vaccine, newspaper articles did not question its safety or effectiveness. Instead the vaccine was described as a needed, desirable, and scarce commodity. In the following paragraphs, we discuss our survey findings in the context of our analysis of the newspaper coverage.

Some interesting commonalities and divergencies were observed between the (changes in) public perceptions and responses on the one hand and the newspaper articles on the other. Firstly, between December 2017 – sometime after the announcement that the government would offer the menACWY vaccination to toddlers and teenagers – and September 2018 – at the start of the first catch-up vaccination rounds – we observed a clear increase in people’s perceived severity of IMD. In this period, the number of articles about IMD increased rapidly, and a majority of these newspaper articles informed people about the severe diseases and symptoms caused by a meningococcal W infection and the possible fatal consequences of an infection. Between September 2018 and July 2019, fewer newspaper articles provided information about the disease symptoms, but more articles discussed narratives on adolescents who had suffered from or died due to IMD. Narratives of personal stories can be highly emotion-laden, and emotion-laden information has been shown to increase perceptions of severity [28]. In this second period, we did not, however, observe a further increase in perceived severity. This might also have been caused by a ceiling effect in the survey measurement.

Secondly, between December 2017 and July 2019, we did not observe considerable increases in people’s perception of the probability of infection even though most newspaper articles reported on the rise in meningococcal W infections and only a small proportion of the articles mentioned that the absolute personal risk of infection was small. Most articles also mentioned mortality and morbidity statistics. People might have correctly interpreted the meaning of the relatively small number of IMD cases in the Netherlands (e.g., 103 in 2018 [1]) for their own and their child’s probability of contracting IMD. Notably, we did observe a clear decrease in the perceived probability of infection of one’s child among parents of teenagers. Most of these teenagers had been vaccinated in the catch-up campaign in that period and thus were indeed less likely to contract the disease.

Thirdly, we observed a steady increase in positive attitudes towards the menACWY vaccination between December 2017 and July 2019. During this period, many newspaper articles discussed the shortage of menACWY vaccinations and the increased demand for these vaccines. In addition, and in contrast to, for example, the introduction of the HPV vaccination for teenage girls [37, 38], the newspaper articles rarely questioned the safety or effectiveness of the menACWY vaccine. Those articles that did discuss some critical notes regarding the vaccination asked whether the vaccination should have been offered earlier or to more age groups. All these content elements suggest a representation of the menACWY vaccine as a needed, desirable, and scarce commodity.

Fourthly, public trust in the RIVM, the government, and pharmaceutical companies increased slightly between December 2017 and July 2019. This increase in trust differs from the situation during the H1N1 epidemic when public trust in various institutions involved in managing the epidemic decreased considerably over time [9]. This decrease in trust during the H1N1 was attributed to, among other things, the public perception that authorities had ulterior (economic) motives for a large-scale vaccination campaign. The public discussion, or at least the newspaper coverage, about the menACWY vaccine was very different in that sense, as the most salient critique was that the vaccination campaign would be implemented too late and not on a large enough scale. In addition, the newspaper articles did not criticize pharmaceutical companies. These organizations were mostly mentioned in the articles as the ones who had warned the RIVM and the government about the rise in infections and the need to start vaccinating.

Finally, between December 2017 and September 2018, the willingness to receive the menACWY vaccination increased in all studied groups, both with regard to their child as themselves. While the willingness to vaccinate their child was stronger in parents of teenagers who had been invited for the menACWY vaccination than in other parents (both overall and in the increase over time before the first vaccination rounds), both parent groups showed considerable willingness to have their child vaccinated in September 2018. The observed high willingness to be, and to have one’s child, vaccinated corresponds with the generally high menACWY vaccination uptake among teenagers (87% in 2018, [2]) and might explain the run on menACWY vaccines in September 2018 ( [2]; see also Fig. 1). The increase in willingness to vaccinate may be related to the increase in perceived severity and positive attitude towards the menACWY vaccination, which are shown determinants of vaccination intentions and behavior [4], and may have been indirectly influenced by the media coverage.

We did not find any notable differences in public perceptions and responses between parents of under-age children and individuals with no under-age children, and we found only a few differences between parents of teenagers invited for the menACWY vaccination and parents of other under-age children. As discussed earlier, parents of teenagers were, compared with other parents, more – and more increasingly – willing to have their child vaccinated against IMD and showed a stronger decrease in the perceived probability of their child contracting IMD after the vaccination rounds. In addition, parents of teenagers perceived the severity of themselves contracting IMD as somewhat higher. One possible explanation for this latter finding may be that parents of teenagers may have felt more involved in the topic and, therefore, were more motivated to adopt information about IMD. This increased motivation consequently may have increased the extent to which parents of teenagers have informed themselves about the severity of IMD [39].

Our study has several limitations. Firstly, our methods did not allow for causal inferences between newspaper coverage and public perceptions and responses. With our results, we can only show similarities and differences between these two dynamics during an outbreak situation and hypothesize about associations between the two based on previous research. Previous research has shown associations between media reporting and public perceptions of risk and of institutions that are responsible for managing that risk [23–29]. We believe that our study provides useful additional insights by providing an integrated portrait of these interrelated social dynamics based on real-life data. A second limitation of this study is that we have only studied the coverage by newspapers regarding the IMD outbreak and have not incorporated other important disseminators of information, such as television and social media but also, importantly, communications from the involved institutions. Nevertheless, we presume that part of these influences was also reflected in the analyzed newspaper articles. Third, while our survey population was largely representative of the Dutch general population in terms of demographic characteristics, we did observe some differences between respondents who participated in all three surveys, and respondents who did not participate in all surveys. Most importantly, the drop-out was somewhat higher among younger and lower educated respondents. In addition, we used an online panel population for data collection, and questions have been raised about whether participants in these panels are sufficiently representative of the general population [40]. Possibly, online survey panel participants differ from the general population in characteristics that cannot be assessed with demographic variables such as age and education. For example, online survey panel populations might be more digital literate and have better access to digital sources compared to the general population. Nevertheless, online survey panels are largely used in research of this kind, being one of the few instruments that can enable rapid implementation of survey studies at limited costs, which is essential when studying the public’s response to an unexpected disease outbreak.

The results of this study suggest that the media can complement public health institutes’ efforts in disseminating their message and encourage protective behavior, in contrast to some studies in which the media seemed mainly a disturbing factor in public health attempts to encourage vaccination behavior [41, 42]. Previous research has also suggested that journalists see it as their responsibility to accurately disseminate information from the authorities during health crises and even to encourage the public to display protective behavior [43]. Our results suggest that during the outbreak of IMD, possibly partly due to the outbreak response and the information provision about it, public perceptions of IMD severity, more positive attitudes to the menACWY vaccination, and higher willingness to be vaccinated with the menACWY have increased. Moreover, there may even have been spill-over effects on public perceptions of vaccinations in general as a change was observed in the trend of overall vaccination coverage in the Netherlands. During the period of the menACWY catch-up vaccination campaign: from 2018 to 2019, the overall NIP vaccination coverage, which had been gradually decreasing since 2014, stabilized [2].

## Conclusion

The real-time insights from this study into the interrelated dynamics of public perceptions, responses, and newspaper coverage provide an integrated portrait of the social developments during the IMD outbreak. Overall, the response to this outbreak and the information provision about this outbreak appeared successful in multiple aspects. The public perceived IMD as severe, appeared aware that the probability of contracting the disease was low despite the rise in infections, had a positive attitude towards the menACWY vaccination, had stable trust in the authorities, and was willing to adopt the menACWY vaccination. Newspaper coverage about this vaccination was largely in line with these public perceptions and responses as it put considerable emphasis on the severity of IMD and provided a representation of the menACWY vaccination as a scarce and desirable commodity to protect against IMD. The focus on IMD severity and the absence of doubt in the public discussion about vaccine safety possibly played an important role in the societal response to this outbreak and the recommended vaccine.

## Supplementary Information

**Additional file 1. Figure S1.** (Number of patients with invasive meningococcal disease caused by different serotypes in the Netherlands, 1992–2019).

**Additional file 2.** The first survey. The second survey. The Third survey.

**Additional file 3. Table S1.** (Differences between respondents who participated in all three survey waves (S1-S3) and respondents who did not participate in all three survey waves. Differences were studied in demographic characteristics (age, sex and education level) and all outcome measures (shown in Table 1 main text) from the first survey (S1)).

**Additional file 4. Table S2.** (Means (M) and standard deviations (SD) of perceptions, trust, and willingness to vaccinate among individuals (NC), parents (T + O), Parents (T) and parents (O). These descriptives include only the respondents who participated in all three survey rounds (T1-T3)). Table 3 (Multilevel analyses in Individuals (NC) and Parents (T + O). All analyses were controlled for age, sex and education level.). Table S4. (Multilevel analyses in Parents (T and O). All analyses were controlled for age, sex and education level.).

**Additional file 5 Table S5**. (Number of newspaper articles about meningococcal W disease and/or the menACWY vaccination per survey period.).

## Data Availability

The datasets used and/or analysed during the current study available from the corresponding author on reasonable request.

## Abbreviations

IMD: Invasive Meningococcal Disease
NIP: National Immunization Program
RIVM: National Institute for Public Health and the Environment in the Netherlands (In Dutch: *Rijksinstituut voor Volksgezondheid en Milieu*)

## Footnotes

1 We only asked these questions with regard to parents’ youngest child, and not separate questions for all their children under the age of 18 years, to prevent survey fatigue (if we would have added questions for all children). We also did not want to generalize by for example only asking about ‘your child(ren)’, as then it would be difficult to distinguish between perceptions regarding those children targeted for a vaccination and those not targeted.

2 The newspapers included were Algemeen Dagblad, Telegraaf, Volkskrant, Nederlands Dagblad, Reformatorisch Dagblad, Trouw, Metro, Financieel Dagblad and NRC Handelsblad. One national newspaper was excluded from the analysis (NRC Next) because of the high overlap in articles with one of the other newspapers (NRC Handelsblad).

3 Voormolen, S. (2018, September 22). The dilemma whether or not to vaccinate (In Dutch: Het dilemma van wel of niet prikken). NRC Handelsblad, p.1.

4 Nieuwenhuis, M. (2018, December 19). “Inoculation against type W useful for all Dutch people” (In Dutch: “Prik tegen type W nuttig voor álle Nederlanders”. Algemeen Dagblad, p.5.

## References

[1] Knol MJ, Ruijs WL, Antonise-Kamp L, de Melker HE, van der Ende A (2018). Implementation of MenACWY vaccination because of ongoing increase in serogroup W invasive meningococcal disease, the Netherlands, 2018. Eurosurveillance.

[2] Baboe Kalpoe S, K S, M, Benschop , B H, B, van Benthem , G A, M, Berbers, R vB, R B (2019). The National Immunisation Programme in the Netherlands: Surveillance and developments in 2018–2019.

[3] Lima POB, van Lier A, de Melker H, Ferreira JA, van Vliet H, Knol MJ (2020). MenACWY vaccination campaign for adolescents in the Netherlands: uptake and its determinants. Vaccine.

[4] Betsch C, Böhm R, Chapman GB (2015). Using behavioral insights to increase vaccination policy effectiveness. Policy Insights Behav Brain Sci.

[5] Gilles I, Bangerter A, Clémence A, Green EG, Krings F, Staerklé C (2011). Trust in medical organizations predicts pandemic (H1N1) 2009 vaccination behavior and perceived efficacy of protection measures in the Swiss public. Eur J Epidemiol.

[6] Reintjes R, Das E, Klemm C, Richardus JH, Keßler V, Ahmad A (2016). “Pandemic public health paradox”: time series analysis of the 2009/10 influenza a/H1N1 epidemiology, media attention, risk perception and public reactions in 5 European countries. PLoS One.

[7] van der Weerd W, Timmermans DR, Beaujean DJ, Oudhoff J, van Steenbergen JE (2011). Monitoring the level of government trust, risk perception and intention of the general public to adopt protective measures during the influenza a (H1N1) pandemic in the Netherlands. BMC Public Health.

[8] Bults M, Beaujean DJ, Richardus JH, Voeten HA (2015). Perceptions and behavioral responses of the general public during the 2009 influenza A (H1N1) pandemic: a systematic review. Disaster Med Public Health Prep.

[9] Bangerter A, Krings F, Mouton A, Gilles I, Green EG, Clémence A (2012). Longitudinal investigation of public trust in institutions relative to the 2009 H1N1 pandemic in Switzerland. PLoS One.

[10] Mayor E, Eicher V, Bangerter A, Gilles I, Clémence A, Green EG (2013). Dynamic social representations of the 2009 H1N1 pandemic: shifting patterns of sense-making and blame. Public Underst Sci.

[11] Bults M, Beaujean DJ, de Zwart O, Kok G, van Empelen P, van Steenbergen JE (2011). Perceived risk, anxiety, and behavioural responses of the general public during the early phase of the influenza a (H1N1) pandemic in the Netherlands: results of three consecutive online surveys. BMC Public Health.

[12] Timmermans DR, Henneman L, Hirasing RA, van der Wal G (2008). Parents' perceived vulnerability and perceived control in preventing Meningococcal C infection: a large-scale interview study about vaccination. BMC Public Health.

[13] Timmermans DR, Henneman L, Hirasing RA, van der Wal G (2005). Attitudes and risk perception of parents of different ethnic backgrounds regarding meningococcal C vaccination. Vaccine.

[14] Dubé E, Gagnon D, Hamel D, Belley S, Gagné H, Boulianne N (2015). Parents’ and adolescents’ willingness to be vaccinated against serogroup B meningococcal disease during a mass vaccination in Saguenay–lac-St-Jean (Quebec). Can J Infect Dis Med Microbiol.

[15] Wang B, Clarke M, Afzali HH, Marshall H (2014). Community, parental and adolescent awareness and knowledge of meningococcal disease. Vaccine.

[16] Trayner KM, Anderson N, Cameron JC (2019). A mixed-methods study to identify factors associated with MenACWY vaccine uptake, barriers and motivations towards vaccination among undergraduate students. Health Educ J.

[17] Blagden S, Seddon D, Hungerford D, Stanistreet D (2017). Uptake of a new meningitis vaccination programme amongst first-year undergraduate students in the United Kingdom: a cross-sectional study. PLoS One.

[18] Tho SLN, Ader F, Ferry T, Floret D, Arnal M, Fargeas S (2015). Vaccination against serogroup B Neisseria meningitidis: perceptions and attitudes of parents. Vaccine.

[19] de Vries M, Claassen L, te Wierik MJ, Coban F, Wong A, Timmermans DR (2020). Meningococcal W 135 disease vaccination intent, the Netherlands, 2018–2019. Emerg Infect Dis.

[20] Breakwell L, Vogt TM, Fleming D, Ferris M, Briere E, Cohn A (2016). Understanding factors affecting university a students' decision to receive an unlicensed serogroup B meningococcal vaccine. J Adolesc Health.

[21] Seeger MW (2006). Best practices in crisis communication: an expert panel process. J Appl Commun Res.

[22] Kasperson RE, Renn O, Slovic P, Brown HS, Emel J, Goble R (1988). The social amplification of risk: a conceptual framework. Risk Anal.

[23] Hong Y, Kim JS, Xiong L (2019). Media exposure and individuals’ emergency preparedness behaviors for coping with natural and human-made disasters. J Environ Psychol.

[24] Zhao M, Rosoff H, John RS (2019). Media disaster reporting effects on public risk perception and response to escalating tornado warnings: a natural experiment. Risk Anal.

[25] Renn O, Burns WJ, Kasperson JX, Kasperson RE, Slovic P. The social amplification of risk: Theoretical foundations and empirical applications. J Soc Issues. 1992;48(4):137–60.

[26] Combs B, Slovic P (1979). Newspaper coverage of causes of death. Journal Q.

[27] Frewer LJ, Miles S, Marsh R (2002). The media and genetically modified foods: evidence in support of social amplification of risk. Risk Anal.

[28] Klemm C, Hartmann T, Das E (2019). Fear-mongering or fact-driven? Illuminating the interplay of objective risk and emotion-evoking form in the response to epidemic news. Health Commun.

[29] Markon M-PL, Lemyre L (2013). Public reactions to risk messages communicating different sources of uncertainty: an experimental test. Human and ecological risk assessment: an. Int J.

[30] Wahlberg AAF, Sjoberg L (2000). Risk perception and the media. J Risk Res.

[31] Lipps O, Herzing JM, Pekari N, Ernst Stähli M, Pollien A, Riedo G, Reveilhac M. Incentives in surveys. FORS Guide No. 08, Version 1.0. Lausanne: Swiss Centre of Sciences FORS; 2019. 10.24449/FG-2019-00008.

[32] CCMO. Your research: Is it Is it subject to the WMO or not? [Available from: https://english.ccmo.nl/investigators/legal-framework-for-medical-scientific-research/your-research-is-it-subject-to-the-wmo-or-not]. Accessed 1 July 2019.

[33] Peters RG, Covello VT, McCallum DB (1997). The determinants of trust and credibility in environmental risk communication: an empirical study. Risk Anal.

[34] Tavakol M, Dennick R (2011). Making sense of Cronbach's alpha. Int J Med Educ.

[35] Lexis Nexis. [Nexis search operator] n.d. [Available from: https://advance.lexis.com]. Accessed 14 Jan 2020.

[36] McHugh ML (2012). Interrater reliability: the kappa statistic. Biochem Med.

[37] De Melker H, Kenter G, Van Rossum T, Conyn-Van SM (2012). Stand van zaken: Ontwikkelingen omtrent de HPV-vaccinatie. Ned Tijdschr Geneeskd.

[38] Rondy M, Van Lier A, Van de Kassteele J, Rust L, De Melker H (2010). Determinants for HPV vaccine uptake in the Netherlands: a multilevel study. Vaccine.

[39] Petty RE, Cacioppo JT (1986). The elaboration likelihood model of persuasion.

[40] Brüggen E, van den Brakel JA, Krosnick J. Establishing the accuracy of online panels for survey research. The Hague: Statistics Netherlands; 2016.

[41] Rachiotis G, Mouchtouri V, Kremastinou J, Gourgoulianis K, Hadjichristodoulou C (2010). Low acceptance of vaccination against the 2009 pandemic influenza a (H1N1) among healthcare workers in Greece. Eurosurveillance.

[42] Tsuda K, Yamamoto K, Leppold C, Tanimoto T, Kusumi E, Komatsu T, Kami M. Trends of media coverage on human papillomavirus vaccination in Japanese newspapers. Clin Infect Dis. 2016:ciw647. 10.1093/cid/ciw647 27660235

[43] Klemm C, Das E, Hartmann T (2019). Changed priorities ahead: journalists’ shifting role perceptions when covering public health crises. Journalism.

