# Supplementary material for: Dynamics in public perceptions and media coverage during an ongoing outbreak of meningococcal W disease in the Netherlands
Source: BMC Public Health. 2022 Apr 1;22:633. doi: 10.1186/s12889-022-12920-8 (PMC8973985; doi:10.1186/s12889-022-12920-8)
Supplement: Supplementary file 1 — Additional file 1. Figure S1. (Number of patients with invasive meningococcal disease caused by different serotypes in the Netherlands, 1992–2019). [file 12889_2022_12920_MOESM1_ESM.docx]

**Supplementary File 1**

*Supplementary File Figure 1. Number of patients with invasive meningococcal disease caused by different serotypes in the Netherlands, 1992-2019 (These figures have been published elsewhere (1)).*

Reference:

1. Pluijmaekers AJM & de Melker HE. *The National Immunisation Programme in the Netherlands. Surveillance and developments in 2020-2021.* Bilthoven: RIVM; 2021.
