# Supplementary material for: Dynamics in public perceptions and media coverage during an ongoing outbreak of meningococcal W disease in the Netherlands
Source: BMC Public Health. 2022 Apr 1;22:633. doi: 10.1186/s12889-022-12920-8 (PMC8973985; doi:10.1186/s12889-022-12920-8)
Supplement: Supplementary file 2 — Additional file 2. The first survey. The second survey. The Third survey. [file 12889_2022_12920_MOESM2_ESM.docx]

**Supplementary File 2**

**First Questionnaire Meningococcal Disease**

** This survey is translated from Dutch, please contact the first author for the original survey in Dutch*

Invitation

This research is conducted by the VU Medical Center Amsterdam in collaboration with the National Institute for Public Health and the Environment (RIVM). With this questionnaire we want to investigate how you think about infectious diseases caused by meningococci. In addition, we will ask you a number of questions about vaccinations, trust in authorities and news items. Filling in the questionnaire takes about 15 minutes. Your participation is voluntary and your answers will remain anonymous. In a few months we would like to invite you again to participate in a follow-up study.

*Page 1*

Verification question

*Page 2*

What are meningococci?

This questionnaire is about meningococci. Meningococci are bacteria that can cause disease. Most of these bacteria reside in the nose and throat of healthy people without making them sick. If the bacteria get into the blood or nervous system, it can lead to serious illnesses such as blood poisoning or meningitis. The bacteria are transmittable from person to person.

There are several types of meningococci. These different types can all lead to the same diseases such as blood poisoning and meningitis. In the Netherlands, children of 14 months old are offered a vaccination to protect against meningococcal type C. Since October 2016, the number of people in the Netherlands who become ill with another type of meningococcus, namely meningococcal type W, rises. The current vaccination against meningococcal C does not protect against meningococcal W.

*Page 3*

1. Before receiving this questionnaire, had you ever heard of meningococci?

⃝ Yes

⃝ No

*Page 4*

The questions in this questionnaire are not about what you know, but about what you think and feel. We would therefore want to ask you not to search for more information about meningococci while completing the questionnaire.

*Page 5*

In order to show you the appropriate questions in this questionnaire, we would like to know whether you have children under the age of 18.

2. Do you have children under the age of 18?

⃝ Yes

⃝ No

**Routing: only if 'yes' at 2*

3. How many children under the age of 18 do you have?

…………………………(add control: min. 1, max. 20)

**based on the answer to this question, it is determined how many questions 4 (a to k) the respondent will see.*

*Page 6*

4a. What is the birth year of your youngest child? …… (min 1998, max 2017)

4b. What is the gender of your youngest child?

⃝ A boy

⃝ A girl

*Page 7*

4c. What is the birth year of your oldest child? …… (min 1998, max 2017)

*Page 8*

4d. What is the birth year of your second child? …… (min 1998, max 2017)

By this we mean the second born child.

*Page 9*

4e. What is the birth year of your third child? …… (min 1998, max 2017)

By this we mean the child who was born third.

*Page 10*

4f. What is the birth year of your fourth child? …… (min 1998, max 2017)

By this we mean the child who is born fourth.

*Page 11*

4g. What is the birth year of your fifth child? …… (min 1998, max 2017)

By this we mean the child who was born fifth.

*Page 12*

4h. What is the birth year of your sixth child? …… (min 1998, max 2017)

By this we mean the child who was born sixth.

*Page 13*

4i. What is the birth year of your seventh child? …… (min 1998, max 2017)

By this we mean the child who was born seventh.

*Page 14*

4y. What is the birth year of your eighth child? …… (min 1998, max 2017)

By this we mean the child who was born eight.

*Page 15*

4k. What is the birth year of your ninth child? …… (min 1998, max 2017)

By this we mean the child who was born ninth.

*Page 16*

*(scroll matrix)*

The following questions are about the health risk of meningococci. We would like to know what you think and feel. So give the answer to these questions that best fits your idea or feeling.

5. Do you think that you may get sick due to meningococci in the following 12 months? In your opinion, how likely is this?

Very unlikely ⃝ ⃝ ⃝ ⃝ ⃝ ⃝ ⃝ Very likely

6. Do you think that you may get sick due to meningococci in your life? In your opinion, how likely is this?

Very unlikely ⃝ ⃝ ⃝ ⃝ ⃝ ⃝ ⃝ Very likely

7. How would it be for you if you got sick due to meningococci?

Not at all severe ⃝ ⃝ ⃝ ⃝ ⃝ ⃝ Very severe

*Page 17 (scroll matrix)*

**Routing: only if 'yes' at 2*

The following questions are about 'your child'. If you have more than one child, we mean your youngest child in these questions. Also for these questions, give the answer that best fits your idea or feeling.

8. Do you think your (youngest) child may get sick due to meningococci in the next 12 months? In your opinion, how likely is this?

Very unlikely ⃝ ⃝ ⃝ ⃝ ⃝ ⃝ ⃝ Very likely

9. Do you think your (youngest) child may get sick due to meningococci in his / her life? In your opinion, how likely is this?

Very unlikely ⃝ ⃝ ⃝ ⃝ ⃝ ⃝ ⃝ very likely

10. How would it be for you if your youngest child got sick due to meningococci?

Not at all severe ⃝ ⃝ ⃝ ⃝ ⃝ ⃝ ⃝ Very severe

*Page 18*

11. Below you will find a number of statements that people use to describe themselves. Read every statement and tick one of the booths to the right of that statement to indicate how you feel if you think of meningococci. There are no good or bad answers. Don't think too long. Give your first impression, which is usually the best.

|  | Not at all | Somewhat | Moderately so | Very much so |
| --- | --- | --- | --- | --- |
| a. I feel calm | ⃝ | ⃝ | ⃝ | ⃝ |
| b. I am tense | ⃝ | ⃝ | ⃝ | ⃝ |
| c. I feel confused | ⃝ | ⃝ | ⃝ | ⃝ |
| d. I feel relaxed | ⃝ | ⃝ | ⃝ | ⃝ |
| e. I feel content | ⃝ | ⃝ | ⃝ | ⃝ |
| f. I am worried | ⃝ | ⃝ | ⃝ | ⃝ |
| G. I am angry | ⃝ | ⃝ | ⃝ | ⃝ |
| h. I am annoyed | ⃝ | ⃝ | ⃝ | ⃝ |

** Based upon: van der Bij, A. K., de Weerd, S., Cikot, R. J., Steegers, E. A., & Braspenning, J. C. (2003). Validation of the dutch short form of the state scale of the Spielberger State-Trait Anxiety Inventory: considerations for usage in screening outcomes. Public Health Genomics, 6(2), 84-87.*

*Page 19*

The following questions are about vaccinations. We would like to know what you think and feel. Please give the answers to these questions that best suit your idea or feeling.

12. Children are offered vaccinations in the Netherlands through the National Immunization Program. These vaccinations are to protect children against infectious diseases. What do you think in general about the National Immunization Program?

I think it is …

a. Unnecessary ⃝ ⃝ ⃝ ⃝ ⃝ ⃝ ⃝ Necessary

b. Acceptable ⃝ ⃝ ⃝ ⃝ ⃝ ⃝ ⃝ Unacceptable

c. Safe ⃝ ⃝ ⃝ ⃝ ⃝ ⃝ ⃝ Dangerous

d. Poor ⃝ ⃝ ⃝ ⃝ ⃝ ⃝ ⃝ Good

e. Not self-evident ⃝ ⃝ ⃝ ⃝ ⃝ ⃝ ⃝ Self-evident

*Page 20*

**Routing: Only as 'yes' at 2.*

13. What do you think about the National Immunization Program if you think of your (youngest) child?

I think it is …

a. Unnecessary ⃝ ⃝ ⃝ ⃝ ⃝ ⃝ ⃝ Necessary

b. Acceptable ⃝ ⃝ ⃝ ⃝ ⃝ ⃝ ⃝ Unacceptable

c. Safe ⃝ ⃝ ⃝ ⃝ ⃝ ⃝ ⃝ Dangerous

d. Poor ⃝ ⃝ ⃝ ⃝ ⃝ ⃝ ⃝ Good

e. Not self-evident ⃝ ⃝ ⃝ ⃝ ⃝ ⃝ ⃝ Self-evident

*Page 21*

**Routing: Only as 'yes' at 2.*

14. Parents can choose whether or not they have their children vaccinated according to the National Immunization Program.

Has your (youngest) child have received all vaccinations so far that were offered to him or her through the National Immunization Program? Please note, we only mean the vaccinations that were offered to your child so far.

⃝ Yes

⃝ No, he/she did not receive one or more of the vaccinations

⃝ No, he/she received none of these vaccinations

⃝ I don't know (anymore)

⃝ My child is still too young for vaccinations / no vaccinations have yet been offered

15. Do you want your (youngest) child to receive all vaccinations that are offered to him or her through the National Immunization Program in the future?

⃝ Yes

⃝ No, one or more of the vaccinations not

⃝ No, none of these vaccinations

⃝ I don't know (yet)

⃝ My child has already received all vaccinations

*Page 22*

We will now show you a news item from the national government dated 25 September 2017. Please read this text carefully.

**Schippers adjusts vaccination against meningococci**

Minister Edith Schippers of Health and Sport has decided to adjust the vaccination against meningococci following the advice from an expert council.

• In the course of 2018, all babies aged 14 months will receive a vaccination that protects against meningococci types A, C, W and Y.

• Young people in lower secondary education will also receive an invitation for a vaccination against meningococci.

With the choice of a combination vaccine, the minister wants to stop the steady increase in infections with meningococcal type W. The number of infections has risen from an average of four per year in the years before 2015, to fifty in 2016, to 47 to the first of August this year.

*Page 23*

16. What do you think in general of the advice of the expert council for vaccination against meningococcal type W?

I think it is …

a. Unnecessary ⃝ ⃝ ⃝ ⃝ ⃝ ⃝ ⃝ Necessary

b. Acceptable ⃝ ⃝ ⃝ ⃝ ⃝ ⃝ ⃝ Unacceptable

c. Safe ⃝ ⃝ ⃝ ⃝ ⃝ ⃝ ⃝ Dangerous

d. Poor ⃝ ⃝ ⃝ ⃝ ⃝ ⃝ ⃝ Good

e. Not self-evident ⃝ ⃝ ⃝ ⃝ ⃝ ⃝ ⃝ Self-evident

*Page 24*

** Routing: Only as 'yes' at 2*

17. What do you think in general of the advice of the expert council for vaccination against meningococcal type W when you think of your (youngest) child?

I think it is …

a. Unnecessary ⃝ ⃝ ⃝ ⃝ ⃝ ⃝ ⃝ Necessary

b. Acceptable ⃝ ⃝ ⃝ ⃝ ⃝ ⃝ ⃝ Unacceptable

c. Safe ⃝ ⃝ ⃝ ⃝ ⃝ ⃝ ⃝ Dangerous

d. Poor ⃝ ⃝ ⃝ ⃝ ⃝ ⃝ ⃝ Good

e. Not self-evident ⃝ ⃝ ⃝ ⃝ ⃝ ⃝ ⃝ Self-evident

*Page 25*

18. Do you mainly see advantages or disadvantages of vaccination against meningococcal type W?

I think there are ...

| Mainly advantages |  |  | As many advantages as disadvantages |  |  | Mainly disadvantages |
| --- | --- | --- | --- | --- | --- | --- |
| ⃝ | ⃝ | ⃝ | ⃝ | ⃝ | ⃝ | ⃝ |

19. If you want, you can explain your answers to this page here.

…………………………………………………………………………………….

…………………………………………………………………………………….

*Page 26*

20. Below you will find a number of statements that people use to describe themselves. Read every judgment and tick one of the booths to the right of that statement to indicate how you feel if you think of vaccination against meningococcal type W. There are no good or bad answers. Don't think too long. Give your first impression, which is usually the best.

Please note that this question is about your feelings regarding vaccination against meningococcal type W, so it is not about your feelings regarding the risk of meningococcal disease.

|  | Not at all | Somewhat | Moderately so | Very much so |
| --- | --- | --- | --- | --- |
| a. I feel calm | ⃝ | ⃝ | ⃝ | ⃝ |
| b. I am tense | ⃝ | ⃝ | ⃝ | ⃝ |
| c. I feel confused | ⃝ | ⃝ | ⃝ | ⃝ |
| d. I feel relaxed | ⃝ | ⃝ | ⃝ | ⃝ |
| e. I feel content | ⃝ | ⃝ | ⃝ | ⃝ |
| f. I am worried | ⃝ | ⃝ | ⃝ | ⃝ |
| G. I am angry | ⃝ | ⃝ | ⃝ | ⃝ |
| h. I am annoyed | ⃝ | ⃝ | ⃝ | ⃝ |

** Based upon: van der Bij, A. K., de Weerd, S., Cikot, R. J., Steegers, E. A., & Braspenning, J. C. (2003). Validation of the dutch short form of the state scale of the Spielberger State-Trait Anxiety Inventory: considerations for usage in screening outcomes. Public Health Genomics, 6(2), 84-87.*

*Page 27*

21. Some people have already been vaccinated against meningococcal type W. The vaccine used for this vaccination is called the meningococcal ACWY vaccine. This is another vaccine than the vaccine against meningococcal type C that has been offered in the National Immunization
Program since 2002.

Are you already vaccinated against meningococcal type W?

⃝ Yes, for a travel abroad

⃝ Yes, following the news on meningococcal type W earlier this year

⃝ Yes, but for another reason ...

⃝ No

⃝ I don't know

**Routing: Only if 'No' or 'I don't know' at 21.*

22. Do you want to be vaccinated against meningococcal type W?

Certainly not ⃝ ⃝ ⃝ ⃝ ⃝ ⃝ ⃝ Certainly yes

*Page 28*

**Routing: Only as 'yes' at 2*

23. Is your (youngest) child already vaccinated against meningococcal type W?

⃝ Yes, for a travel abroad

⃝ Yes, following reporting on meningococcal type w earlier this year

⃝ Yes, but for another reason ...

⃝ No

⃝ I don't know

*Page 29*

**Routing: Only as 'yes' at 2 and 'no' or 'I don't know' in question 23.*

24. Do you want your youngest child to be vaccinated against meningococcal disease type A, C, W, and Y?

Certainly not ⃝ ⃝ ⃝ ⃝ ⃝ ⃝ ⃝ Certainly yes

*Page 30*

The following questions are about your trust in different authorities.

25. The Dutch government determines the policy for infectious diseases and vaccinations. What do you think about our government when it comes to infectious diseases and vaccinations? Indicate to what extent you agree or disagree with the statements.

|  | Completely disagree | Disagree | Do not agree nor disagree | Agree | Completely agree |
| --- | --- | --- | --- | --- | --- |
| The government has sufficient knowledge and skills with regard to infectious diseases and vaccinations. | ⃝ | ⃝ | ⃝ | ⃝ | ⃝ |
| The government communicates openly about infectious diseases and vaccinations. | ⃝ | ⃝ | ⃝ | ⃝ | ⃝ |
| The government puts the health of citizens above economic interests when it comes to vaccinations. | ⃝ | ⃝ | ⃝ | ⃝ | ⃝ |

*Page 31*

26. The National Institute for Public Health and the Environment (RIVM) keeps an eye on the risks to public health posed by infectious diseases. Furthermore, the RIVM implements the policy of the government regarding infectious diseases and vaccinations. What do you think about the RIVM when it comes to infectious diseases and vaccinations? Indicate to what extent you agree or disagree with the statements.

|  | Completely disagree | Disagree | Do not agree nor disagree | Agree | Completely agree |
| --- | --- | --- | --- | --- | --- |
| The RIVM has sufficient knowledge and skills with regard to infectious diseases and vaccinations. | ⃝ | ⃝ | ⃝ | ⃝ | ⃝ |
| The RIVM communicates openly about infectious diseases and vaccinations. | ⃝ | ⃝ | ⃝ | ⃝ | ⃝ |
| The RIVM puts the health of citizens above economic interests when it comes to vaccinations. | ⃝ | ⃝ | ⃝ | ⃝ | ⃝ |

*Page 32*

27. Vaccines against infectious diseases are made and sold by pharmaceutical companies. What do you think about pharmaceutical companies when it comes to vaccines? Indicate to what extent you agree or disagree with the statements.

|  | Completely disagree | Disagree | Do not agree nor disagree | Agree | Completely agree |
| --- | --- | --- | --- | --- | --- |
| Pharmaceutical companies have sufficient knowledge and skills with regard to infectious diseases and vaccinations. | ⃝ | ⃝ | ⃝ | ⃝ | ⃝ |
| Pharmaceutical companies communicate openly about infectious diseases and vaccinations. | ⃝ | ⃝ | ⃝ | ⃝ | ⃝ |
| Pharmaceutical companies put the health of citizens above economic interests when it comes to vaccinations. | ⃝ | ⃝ | ⃝ | ⃝ | ⃝ |

*Page 33*

The last questions are not about infectious diseases or vaccinations, but about the news. We would like to know how you keep yourself up date with the news.

28. Indicate which of the media below you consulted in the past 7 days for news items. You can choose multiple answers.

⃝ a. A newspaper (newspaper / website of newspaper / mobile app from newspaper)

⃝ b. Another type of news website or mobile app (for example nu.nl or nos.nl)

⃝ c. A news program (for example RTL Niews or NOS Journaal)

⃝ d. A current affairs program (for example De Wereld Draait Door or RTL Late Night)

⃝ e. News from Social Media (for example via Facebook or Instagram)

⃝ f. Otherwise, namely …

⃝ G. Not applicable, I don’t keep up to date with the news

**Routing: if 'a' ticked at 28:*

29. From which newspaper or newspapers did you read news messages in the past week?

…………………………………………………………………………………….

…………………………………………………………………………………….

**Routing: if 'b' ticked at 28:*

30. From which news website (s) or mobile app (s) did you read news messages in the past week?

…………………………………………………………………………………….

…………………………………………………………………………………….

**Routing: if 'c' ticked at 28:*

31. Which news programs did you view in the past week?

…………………………………………………………………………………….

…………………………………………………………………………………….

**Routing: if 'd' ticked at 28:*

32. What current affairs programs did you view in the past week?

…………………………………………………………………………………….

…………………………………………………………………………………….

*Page 34*

This is the end of the questionnaire. Thank you for you participation!

**Second questionnaire meningococcal disease**

** This survey is translated from Dutch, please contact the first author for the original survey in Dutch*

Invitation

In December last year you participated in a questionnaire about infectious diseases caused by meningococci. In addition, we asked you a number of questions about vaccinations, trust in institutions and news items. This is a follow-up to that questionnaire. We would like to know how you are currently thinking about the aforementioned topics. Completing the questionnaire takes approximately 10-15 minutes. Your participation is voluntary and your answers remain anonymous. In a few months we will invite you one last time to fill in a questionnaire.

This research is conducted by the VU Medical Center Amsterdam in collaboration with the National Institute for Public Health and the Environment (RIVM).

*Page 1*

Verification question

*Page 2*

What are meningococci?

This questionnaire is about meningococci. Meningococci are bacteria that can cause disease. Most of these bacteria reside in the nose and throat of healthy people without making them sick. If the bacteria get into the blood or nervous system, it can lead to serious illnesses such as blood poisoning or meningitis. The bacteria are transmittable from person to person.

There are several types of meningococci. These different types can all lead to the same diseases such as blood poisoning and meningitis. In the Netherlands, children of 14 months old are offered a vaccination to protect against meningococcal type C. Since October 2016, the number of people in the Netherlands who become ill with another type of meningococcus rises, namely meningococcal type W. This is why children are now invited for a vaccination against multiple types of meningococci, instead of the vaccination against only meningococcal type C. Children are now invited for a vaccination against meningococcal types A, C, W and Y.

Please note that if you have had experience with meningococcal disease, some questions in this questionnaire can be experienced as unpleasant.

*Page 3*

The questions in this questionnaire are not about what you know, but about what you think and feel. We would therefore want to ask you not to search for more information about meningococci while completing the questionnaire.

*Page 4*

*(scroll matrix)*

The following questions are about the health risk of meningococci. We would like to know what you think and feel. So give the answer to these questions that best fits your idea or feeling.

1. Do you think that you may get sick due to meningococci in the following 12 months? In your opinion, how likely is this?

Very unlikely ⃝ ⃝ ⃝ ⃝ ⃝ ⃝ ⃝ Very likely

2. Do you think that you may get sick due to meningococci in your life? In your opinion, how likely is this?

Very unlikely ⃝ ⃝ ⃝ ⃝ ⃝ ⃝ ⃝ Very likely

3. How would it be for you if you got sick due to meningococci?

Not at all severe ⃝ ⃝ ⃝ ⃝ ⃝ ⃝ Very severe

*Page 5 (scroll matrix)*

**Routing: only if respondent has (a) child(ren) under the age of 18 years*

The following questions are about 'your child'. If you have more than one child, we mean your youngest child in these questions. Also for these questions, give the answer that best fits your idea or feeling.

4. Do you think your (youngest) child may get sick due to meningococci in the next 12 months? In your opinion, how likely is this?

Very unlikely ⃝ ⃝ ⃝ ⃝ ⃝ ⃝ ⃝ Very likely

5. Do you think your (youngest) child may get sick due to meningococci in his / her life? In your opinion, how likely is this?

Very unlikely ⃝ ⃝ ⃝ ⃝ ⃝ ⃝ ⃝ very likely

6. How would it be for you if your youngest child got sick due to meningococci?

Not at all severe ⃝ ⃝ ⃝ ⃝ ⃝ ⃝ ⃝ Very severe

*Page 6*

7. Below you will find a number of statements that people use to describe themselves. Read every statement and tick one of the booths to the right of that statement to indicate how you feel if you think of meningococci. There are no good or bad answers. Don't think too long. Give your first impression, which is usually the best.

If I think about meningococcal disease…

|  | Not at all | Somewhat | Moderately so | Very much so |
| --- | --- | --- | --- | --- |
| a. I feel calm | ⃝ | ⃝ | ⃝ | ⃝ |
| b. I am tense | ⃝ | ⃝ | ⃝ | ⃝ |
| c. I feel confused | ⃝ | ⃝ | ⃝ | ⃝ |
| d. I feel relaxed | ⃝ | ⃝ | ⃝ | ⃝ |
| e. I feel content | ⃝ | ⃝ | ⃝ | ⃝ |
| f. I am worried | ⃝ | ⃝ | ⃝ | ⃝ |
| G. I am angry | ⃝ | ⃝ | ⃝ | ⃝ |
| h. I am annoyed | ⃝ | ⃝ | ⃝ | ⃝ |

** Based upon: van der Bij, A. K., de Weerd, S., Cikot, R. J., Steegers, E. A., & Braspenning, J. C. (2003). Validation of the dutch short form of the state scale of the Spielberger State-Trait Anxiety Inventory: considerations for usage in screening outcomes. Public Health Genomics, 6(2), 84-87.*

*Page 7*

The following questions are about vaccinations. We would like to know what you think and feel. Please give the answer to these questions that best suits your idea or feeling.

8. Children are offered vaccinations in the Netherlands through the National Immunization Program. These vaccinations are to protect children against infectious diseases. What do you think in general about the National Immunization Program?

I think it is …

a. Unnecessary ⃝ ⃝ ⃝ ⃝ ⃝ ⃝ ⃝ Necessary

b. Acceptable ⃝ ⃝ ⃝ ⃝ ⃝ ⃝ ⃝ Unacceptable

c. Safe ⃝ ⃝ ⃝ ⃝ ⃝ ⃝ ⃝ Dangerous

d. Poor ⃝ ⃝ ⃝ ⃝ ⃝ ⃝ ⃝ Good

e. Not self-evident ⃝ ⃝ ⃝ ⃝ ⃝ ⃝ ⃝ Self-evident

*Page 8*

** Routing: only if respondent has (a) child(ren) under the age of 18 years*

9. What do you think about the National Immunization Program when you think of your (youngest) child?

I think it's …

a. Unnecessary ⃝ ⃝ ⃝ ⃝ ⃝ ⃝ ⃝ Necessary

b. Acceptable ⃝ ⃝ ⃝ ⃝ ⃝ ⃝ ⃝ Unacceptable

c. Safe ⃝ ⃝ ⃝ ⃝ ⃝ ⃝ ⃝ Dangerous

d. Poor ⃝ ⃝ ⃝ ⃝ ⃝ ⃝ ⃝ Good

e. Not self-evident ⃝ ⃝ ⃝ ⃝ ⃝ ⃝ ⃝ Self-evident

*Page 9*

We will now show you from a news item from last year, September 25, 2017. Please read this text carefully.

**Schippers adjusts vaccination against meningococci**

Minister Edith Schippers of Health and Sport has decided to adjust the vaccination against meningococci following the advice from an expert council.

• In the course of 2018, all babies of 14 months old receive a vaccination that protects against meningococci types A, C, W and Y.

• Young people in lower secondary education will also receive an invitation for a vaccination against meningococci.

With the choice of a combination vaccine, the minister wants to stop the steady increase in infections with meningococcal type W. The number of infections has risen from an average of 4 per year in the years before 2015, to 50 in 2016, to 47 to the first of August this year.

*Page 10*

10. What do you think in general of the advice of the expert council for vaccination against meningococcal type W?

I think it's …

a. Unnecessary ⃝ ⃝ ⃝ ⃝ ⃝ ⃝ ⃝ Necessary

b. Acceptable ⃝ ⃝ ⃝ ⃝ ⃝ ⃝ ⃝ Unacceptable

c. Safe ⃝ ⃝ ⃝ ⃝ ⃝ ⃝ ⃝ Dangerous

d. Poor ⃝ ⃝ ⃝ ⃝ ⃝ ⃝ ⃝ Good

e. Not self-evident ⃝ ⃝ ⃝ ⃝ ⃝ ⃝ ⃝ Self-evident

*Page 11*

** Routing: only if respondent has (a) child(ren) under the age of 18 years*

11. What do you think in general of the advice of the expert council for vaccination against meningococcal type W when you think of your (youngest) child?

I think it's …

a. Unnecessary ⃝ ⃝ ⃝ ⃝ ⃝ ⃝ ⃝ Necessary

b. Acceptable ⃝ ⃝ ⃝ ⃝ ⃝ ⃝ ⃝ Unacceptable

c. Safe ⃝ ⃝ ⃝ ⃝ ⃝ ⃝ ⃝ Dangerous

d. Poor ⃝ ⃝ ⃝ ⃝ ⃝ ⃝ ⃝ Good

e. Not self-evident ⃝ ⃝ ⃝ ⃝ ⃝ ⃝ ⃝ Self-evident

*Page 12*

We will now show you a text from a recent news item of July 16, 2018. Please read this text carefully.

**Blokhuis announces large-scale extra vaccination: More than half a million children receive an invitation**

Next year, an additional 650,000 children will be called upon to be vaccinated against the very serious infectious disease meningococcus. This concerns children born between 1 January 2001 and May 2004. State Secretary Paul Blokhuis (VWS) announced this in a letter to the House of Representatives. The reason for this measure an increase in the number of people contracting the meningococcal type W infection in recent years. The extra program is in addition to the already planned meningococcal vaccination of children born between April 30, 2004 and December 31, 2005. The aim is to provide vaccination against meningococcal A, C, W and Y to all 14 to 18-year-olds by the end of 2019.

*Page 13*

12. This year or next year, children born in 2001 to 2005 will receive an invitation for a vaccination against meningococcal types A, C, W and Y. What do you think about this decision in general?

I think it is…

a. Unnecessary ⃝ ⃝ ⃝ ⃝ ⃝ ⃝ ⃝ Necessary

b. Acceptable ⃝ ⃝ ⃝ ⃝ ⃝ ⃝ ⃝ Unacceptable

c. Safe ⃝ ⃝ ⃝ ⃝ ⃝ ⃝ ⃝ Dangerous

d. Poor ⃝ ⃝ ⃝ ⃝ ⃝ ⃝ ⃝ Good

e. Not self-evident ⃝ ⃝ ⃝ ⃝ ⃝ ⃝ ⃝ Self-evident

*Page 14*

** Routing: only if respondent has (a) child(ren) under the age of 18 years*

13. This year or next year, children born in 2001 to 2005 will receive an invitation for a vaccination against meningococcal types A, C, W and Y. What do you think about this decision, when you think about your youngest child?

I think it is…

a. Unnecessary ⃝ ⃝ ⃝ ⃝ ⃝ ⃝ ⃝ Necessary

b. Acceptable ⃝ ⃝ ⃝ ⃝ ⃝ ⃝ ⃝ Unacceptable

c. Safe ⃝ ⃝ ⃝ ⃝ ⃝ ⃝ ⃝ Dangerous

d. Poor ⃝ ⃝ ⃝ ⃝ ⃝ ⃝ ⃝ Good

e. Not self-evident ⃝ ⃝ ⃝ ⃝ ⃝ ⃝ ⃝ Self-evident

14. Do you mainly see advantages or disadvantages of vaccination against meningococcal types A, C, W and Y?

I think there are ...

| Mainly advantages |  |  | As many advantages as disadvantages |  |  | Mainly disadvantages |
| --- | --- | --- | --- | --- | --- | --- |
| ⃝ | ⃝ | ⃝ | ⃝ | ⃝ | ⃝ | ⃝ |

15. If you wish, you can explain here your answers to the questions about the decision for vaccination against meningococcal types A, C, W and Y.

…………………………………………………………………………………….

…………………………………………………………………………………….

*Page 16*

16. Below you will find a number of statements that people use to describe themselves. Read every judgment and tick one of the booths to the right of that statement to indicate how you feel if you think of vaccination against meningococcal types A, C, W and Y. There are no good or bad answers. Don't think too long. Give your first impression, which is usually the best.

Please note that this question is about your feelings regarding vaccination against meningococcal types A, C, W and Y, so it is not about your feelings regarding the risk of meningococcal disease.

If I think about the meningococcal vaccination …

|  | Not at all | Somewhat | Moderately so | Very much so |
| --- | --- | --- | --- | --- |
| a. I feel calm | ⃝ | ⃝ | ⃝ | ⃝ |
| b. I am tense | ⃝ | ⃝ | ⃝ | ⃝ |
| c. I feel confused | ⃝ | ⃝ | ⃝ | ⃝ |
| d. I feel relaxed | ⃝ | ⃝ | ⃝ | ⃝ |
| e. I feel content | ⃝ | ⃝ | ⃝ | ⃝ |
| f. I am worried | ⃝ | ⃝ | ⃝ | ⃝ |
| G. I am angry | ⃝ | ⃝ | ⃝ | ⃝ |
| h. I am annoyed | ⃝ | ⃝ | ⃝ | ⃝ |

** Based upon: van der Bij, A. K., de Weerd, S., Cikot, R. J., Steegers, E. A., & Braspenning, J. C. (2003). Validation of the dutch short form of the state scale of the Spielberger State-Trait Anxiety Inventory: considerations for usage in screening outcomes. Public Health Genomics, 6(2), 84-87.*

*Page 17*

17. Some people have already been vaccinated against meningococcal types A, C, W and Y. This is a different vaccination than the meningococcal vaccination that was offered to babies between 2002 and May 2018. That vaccination only protected against meningococci type C.

Have you already been vaccinated against meningococcal types A, C, W and Y?

⃝ Yes, for a foreign trip (info button: the vaccination is advised for travel to Saudi Arabia, Mongolia and parts of Africa)

⃝ Yes, following the news reports about meningococci earlier this year

⃝ Yes, but for a different reason…

⃝ No

⃝ I don't know

**Routing: Only if 'No' or 'I don't know' at 17.*

18. Do you want to be vaccinated against meningococcal type W?

Certainly not ⃝ ⃝ ⃝ ⃝ ⃝ ⃝ ⃝ Certainly yes

*Page 18*

**Routing: only if the respondent has (a) child(ren) under the age of 18 years*

19. Has your (youngest) child already been vaccinated against meningococcal types A, C, W and Y?

⃝ Yes, for a travel abroad (info button: the vaccination is advised for travel to Saudi Arabia, Mongolia and parts of Africa)

⃝ Yes, following the news reports about meningococci earlier this year

⃝ Yes, but for a different reason…

⃝ No

⃝ I don't know

*Page 19*

**Routing: only if the respondent has (a) child(ren) under the age of 18 years and 'No' or 'I don't know' for question 19.*

20. Do you want your youngest child to be vaccinated against meningococcal disease type A, C, W, and Y?

Certainly not ⃝ ⃝ ⃝ ⃝ ⃝ ⃝ ⃝ Certainly yes

*Page 20*

**Routing: only if the respondent has (a) child(ren) under the age of 18 years*

21. All children are offered vaccinations against infectious diseases through the National Immunization Program (NIP). Will your child receive the vaccinations according to this program?

⃝ Yes, my child has received all vaccinations that my child could receive for his/her age from the National Immunization Program

⃝ Yes, partly. My child started vaccinating later and/or did not receive all the vaccinations that my child could receive for his/her age from the National Immunization Program

⃝ No, my child does not participate in the National Immunization Program

⃝ I don't know

⃝ Other, namely…

*Page 21*

The following questions are about your trust in different authorities.

22. The Dutch government determines the policy for infectious diseases and vaccinations. What do you think about our government when it comes to infectious diseases and vaccinations? Indicate to what extent you agree or disagree with the statements.

|  | Completely disagree | Disagree | Do not agree nor disagree | Agree | Completely agree |
| --- | --- | --- | --- | --- | --- |
| The government has sufficient knowledge and skills with regard to infectious diseases and vaccinations. | ⃝ | ⃝ | ⃝ | ⃝ | ⃝ |
| The government communicates openly about infectious diseases and vaccinations. | ⃝ | ⃝ | ⃝ | ⃝ | ⃝ |
| The government puts the health of citizens above economic interests when it comes to vaccinations. | ⃝ | ⃝ | ⃝ | ⃝ | ⃝ |

*Page 22*

23. The National Institute for Public Health and the Environment (RIVM) keeps an eye on the risks to public health posed by infectious diseases. Furthermore, the RIVM implements the policy of the government regarding infectious diseases and vaccinations. What do you think about the RIVM when it comes to infectious diseases and vaccinations? Indicate to what extent you agree or disagree with the statements.

|  | Completely disagree | Disagree | Do not agree nor disagree | Agree | Completely agree |
| --- | --- | --- | --- | --- | --- |
| The RIVM has sufficient knowledge and skills with regard to infectious diseases and vaccinations. | ⃝ | ⃝ | ⃝ | ⃝ | ⃝ |
| The RIVM communicates openly about infectious diseases and vaccinations. | ⃝ | ⃝ | ⃝ | ⃝ | ⃝ |
| The RIVM puts the health of citizens above economic interests when it comes to vaccinations. | ⃝ | ⃝ | ⃝ | ⃝ | ⃝ |

*Page 23*

24. Vaccines against infectious diseases are made and sold by pharmaceutical companies. What do you think about pharmaceutical companies when it comes to vaccines? Indicate to what extent you agree or disagree with the statements.

|  | Completely disagree | Disagree | Do not agree nor disagree | Agree | Completely agree |
| --- | --- | --- | --- | --- | --- |
| Pharmaceutical companies have sufficient knowledge and skills with regard to infectious diseases and vaccinations. | ⃝ | ⃝ | ⃝ | ⃝ | ⃝ |
| Pharmaceutical companies communicate openly about infectious diseases and vaccinations. | ⃝ | ⃝ | ⃝ | ⃝ | ⃝ |
| Pharmaceutical companies put the health of citizens above economic interests when it comes to vaccinations. | ⃝ | ⃝ | ⃝ | ⃝ | ⃝ |

*Page 24*

The last questions are not about infectious diseases or vaccinations, but about the news. We would like to know how you keep up date with the news.

25. Which newspapers, news sites and/or news apps do you regularly read news items from? You can select multiple answers.

⃝ Algemeen Dagblad (AD)

⃝ Blendle

⃝ De Correspondent

⃝ Financieel Dagblad (FD)

⃝ Freedly

⃝ Geen Stijl

⃝ Google nieuws

⃝ Metro

⃝ MSN nieuws

⃝ NOS

⃝ NRC Handelsblad

⃝ NRC Next

⃝ NU.nl

⃝ Reformatorisch Dagblad (RD) / Erdee

⃝ Telegraaf

⃝ Teletekst

⃝ Topics

⃝ Trouw

⃝ Volkskrant (VK)

⃝ Vrouw

⃝ Foreign newspaper(s), such as The Guardian, New York Times, Le Monde

⃝ Regional or local newspaper(s), such as Eindhovens Dagblad, Dagblad van het Noorden, Stentor

⃝ Other, namely….

⃝ I don't read news items

*Page 25*

26. Which news programs and/or current affairs programs do you watch regularly? You can select multiple answers.

⃝ Achter het nieuws

⃝ Brandpunt

⃝ De Wereld Draait Door (DWDD)

⃝ Editie NL

⃝ EenVandaag

⃝ Goedemorgen Nederland

⃝ Hart van Nederland

⃝ Jeugdjournaal

⃝ Jinek

⃝ M

⃝ Monitor

⃝ Nieuwsuur

⃝ NOS journaal

⃝ Pauw

⃝ Radar

⃝ RTL Boulevard

⃝ RTL Late night

⃝ RTL nieuws

⃝ RTLZ nieuws

⃝ RTL ontbijtnieuws

⃝ Show nieuws

⃝ Tegenlicht

⃝ Zembla

⃝ 2doc

⃝ Foreign news programs, such as BBC, Asianews, El Jazeera, Eén

⃝ Local news, such as RTV Drenthe nieuws, Brabant nieuws, TV Noord nieuws

⃝ Other, namely…

⃝ I do not watch news programs or current affairs programs

*Page 26*

This is the end of the questionnaire. Thank you for your participation!

**Third Questionnaire Meningococcal Disease**

** This survey is translated from Dutch, please contact the first author for the original survey in Dutch*

Invitation

At the end of 2017 and in the autumn of 2018, you participated in a questionnaire about infectious diseases caused by meningococci. We also asked you a number of questions about vaccinations and your trust in authorities. This is the last questionnaire of this study. We would like to know what you currently think about the aforementioned topics. Filling in the questionnaire takes about 10-15 minutes. Your participation is voluntary and your answers will remain anonymous.

This research is conducted by the VU Medical Center Amsterdam in collaboration with the National Institute for Public Health and the Environment (RIVM).

*Page 1*

Verification question

*Page 2*

What are meningococci?

This questionnaire is about meningococci. Meningococci are bacteria that can cause disease. Most of these bacteria reside in the nose and throat of healthy people without making them sick. If the bacteria get into the blood or nervous system, it can lead to serious illnesses such as blood poisoning or meningitis. The bacteria are transmittable from person to person.

There are several types of meningococci. These different types can all lead to the same diseases such as blood poisoning and meningitis. In the Netherlands, children of 14 months old are offered a vaccination to protect against meningococcal type C. Since October 2016, the number of people in the Netherlands who become ill with another type of meningococcus rises, namely meningococcal type W. This is why children are now invited for a vaccination against multiple types of meningococci, instead of the vaccination against only meningococcal type C. Children are now invited for a vaccination against meningococcal types A, C, W and Y.

Please note that if you have had experience with meningococcal disease, some questions in this questionnaire can be experienced as unpleasant.

*Page 3*

The questions in this questionnaire are not about what you know, but about what you think and feel. We would therefore want to ask you not to search for more information about meningococci while completing the questionnaire.

*Page 4*

*(scroll matrix)*

The following questions are about the health risk of meningococci. We would like to know what you think and feel. So give the answer to these questions that best fits your idea or feeling.

1. Do you think that you may get sick due to meningococci in the following 12 months? In your opinion, how likely is this?

Very unlikely ⃝ ⃝ ⃝ ⃝ ⃝ ⃝ ⃝ Very likely

2. Do you think that you may get sick due to meningococci in your life? In your opinion, how likely is this?

Very unlikely ⃝ ⃝ ⃝ ⃝ ⃝ ⃝ ⃝ Very likely

3. How would it be for you if you got sick due to meningococci?

Not at all severe ⃝ ⃝ ⃝ ⃝ ⃝ ⃝ Very severe

*Page 5 (scroll matrix)*

**Routing: only if respondent has (a) child(ren) under the age of 18 years*

The following questions are about 'your child'. If you have more than one child, we mean your youngest child in these questions. Also for these questions, give the answer that best fits your idea or feeling.

4. Do you think your (youngest) child may get sick due to meningococci in the next 12 months? In your opinion, how likely is this?

Very unlikely ⃝ ⃝ ⃝ ⃝ ⃝ ⃝ ⃝ Very likely

5. Do you think your (youngest) child may get sick due to meningococci in his / her life? In your opinion, how likely is this?

Very unlikely ⃝ ⃝ ⃝ ⃝ ⃝ ⃝ ⃝ very likely

6. How would it be for you if your youngest child got sick due to meningococci?

Not at all severe ⃝ ⃝ ⃝ ⃝ ⃝ ⃝ ⃝ Very severe

*Page 6*

7. Below you will find a number of statements that people use to describe themselves. Read every statement and tick one of the booths to the right of that statement to indicate how you feel if you think of meningococci. There are no good or bad answers. Don't think too long. Give your first impression, which is usually the best.

If I think about meningococcal disease…

|  | Not at all | Somewhat | Moderately so | Very much so |
| --- | --- | --- | --- | --- |
| a. I feel calm | ⃝ | ⃝ | ⃝ | ⃝ |
| b. I am tense | ⃝ | ⃝ | ⃝ | ⃝ |
| c. I feel confused | ⃝ | ⃝ | ⃝ | ⃝ |
| d. I feel relaxed | ⃝ | ⃝ | ⃝ | ⃝ |
| e. I feel content | ⃝ | ⃝ | ⃝ | ⃝ |
| f. I am worried | ⃝ | ⃝ | ⃝ | ⃝ |
| G. I am angry | ⃝ | ⃝ | ⃝ | ⃝ |
| h. I am annoyed | ⃝ | ⃝ | ⃝ | ⃝ |

** Based upon: van der Bij, A. K., de Weerd, S., Cikot, R. J., Steegers, E. A., & Braspenning, J. C. (2003). Validation of the dutch short form of the state scale of the Spielberger State-Trait Anxiety Inventory: considerations for usage in screening outcomes. Public Health Genomics, 6(2), 84-87.*

*Page 7*

The following questions are about vaccinations. We would like to know what you think and feel. Please give the answer to these questions that best suits your thoughts or feelings.

8. Children are offered vaccinations in the Netherlands through the National Immunization Program. These vaccinations are to protect children against infectious diseases. What do you think in general about the National Immunization Program?

I think it is …

a. Unnecessary ⃝ ⃝ ⃝ ⃝ ⃝ ⃝ ⃝ Necessary

b. Acceptable ⃝ ⃝ ⃝ ⃝ ⃝ ⃝ ⃝ Unacceptable

c. Safe ⃝ ⃝ ⃝ ⃝ ⃝ ⃝ ⃝ Dangerous

d. Poor ⃝ ⃝ ⃝ ⃝ ⃝ ⃝ ⃝ Good

e. Not self-evident ⃝ ⃝ ⃝ ⃝ ⃝ ⃝ ⃝ Self-evident

*Page 8*

** Routing: only if respondent has (a) child(ren) under the age of 18 years*

9. What do you think about the National Immunization Program when you think of your (youngest) child?

I think it is …

a. Unnecessary ⃝ ⃝ ⃝ ⃝ ⃝ ⃝ ⃝ Necessary

b. Acceptable ⃝ ⃝ ⃝ ⃝ ⃝ ⃝ ⃝ Unacceptable

c. Safe ⃝ ⃝ ⃝ ⃝ ⃝ ⃝ ⃝ Dangerous

d. Poor ⃝ ⃝ ⃝ ⃝ ⃝ ⃝ ⃝ Good

e. Not self-evident ⃝ ⃝ ⃝ ⃝ ⃝ ⃝ ⃝ Self-evident

*Page 9*

We will now show you a text. Please read this text carefully.

Since 2015, there has been an increase in people becoming ill with meningococcal type W. The number of infections in the Netherlands has increased from an average of 4 per year in the years before 2015, to 104 per year in 2018. That is why toddlers and teenagers receive a vaccination that protects against meningococcal types A, C, W and Y.

• Since 2018, all 14-month-olds and all 14-year-olds are offered a vaccination that protects against meningococcal types A, C, W and Y.

• In 2018 and 2019, teenagers between the ages of 14 and 18 years received an invitation for a catch-up vaccination against meningococcal types A, C, W and Y. These are teenagers who were born in 2001 to 2005.

*Page 10*

10. In 2018 and 2019, children born in 2001 to 2005 received an invitation for a vaccination against meningococcal types A, C, W and Y. What do you think about this decision in general?

I think it is …

a. Unnecessary ⃝ ⃝ ⃝ ⃝ ⃝ ⃝ ⃝ Necessary

b. Acceptable ⃝ ⃝ ⃝ ⃝ ⃝ ⃝ ⃝ Unacceptable

c. Safe ⃝ ⃝ ⃝ ⃝ ⃝ ⃝ ⃝ Dangerous

d. Poor ⃝ ⃝ ⃝ ⃝ ⃝ ⃝ ⃝ Good

e. Not self-evident ⃝ ⃝ ⃝ ⃝ ⃝ ⃝ ⃝ Self-evident

*Page 11*

** Routing: only if respondent has (a) child(ren) under the age of 18 years*

11. In 2018 and 2019, children born in 2001 to 2005 received an invitation for a vaccination against meningococcal types A, C, W and Y. What do you think about this decision when you think of your (youngest) child?

I think it is …

a. Unnecessary ⃝ ⃝ ⃝ ⃝ ⃝ ⃝ ⃝ Necessary

b. Acceptable ⃝ ⃝ ⃝ ⃝ ⃝ ⃝ ⃝ Unacceptable

c. Safe ⃝ ⃝ ⃝ ⃝ ⃝ ⃝ ⃝ Dangerous

d. Poor ⃝ ⃝ ⃝ ⃝ ⃝ ⃝ ⃝ Good

e. Not self-evident ⃝ ⃝ ⃝ ⃝ ⃝ ⃝ ⃝ Self-evident

*Page 12*

12. Do you mainly see advantages or disadvantages of vaccination against meningococcal types A, C, W and Y?

I think there are ...

| Mainly advantages |  |  | As many advantages as disadvantages |  |  | Mainly disadvantages |
| --- | --- | --- | --- | --- | --- | --- |
| ⃝ | ⃝ | ⃝ | ⃝ | ⃝ | ⃝ | ⃝ |

13. If you wish, you can explain here your answers to the questions about the decision for vaccination against meningococcal types A, C, W and Y.

…………………………………………………………………………………….

…………………………………………………………………………………….

*Page 13*

14. Below you will find a number of statements that people use to describe themselves. Read every judgment and tick one of the booths to the right of that statement to indicate how you feel if you think of vaccination against meningococcal types A, C, W and Y. There are no good or bad answers. Don't think too long. Give your first impression, which is usually the best.

Please note that this question is about your feelings regarding vaccination against meningococcal types A, C, W and Y, so it is not about your feelings regarding the risk of meningococcal disease.

If I think about the meningococcal vaccination …

|  | Not at all | Somewhat | Moderately so | Very much so |
| --- | --- | --- | --- | --- |
| a. I feel calm | ⃝ | ⃝ | ⃝ | ⃝ |
| b. I am tense | ⃝ | ⃝ | ⃝ | ⃝ |
| c. I feel confused | ⃝ | ⃝ | ⃝ | ⃝ |
| d. I feel relaxed | ⃝ | ⃝ | ⃝ | ⃝ |
| e. I feel content | ⃝ | ⃝ | ⃝ | ⃝ |
| f. I am worried | ⃝ | ⃝ | ⃝ | ⃝ |
| G. I am angry | ⃝ | ⃝ | ⃝ | ⃝ |
| h. I am annoyed | ⃝ | ⃝ | ⃝ | ⃝ |

** Based upon: van der Bij, A. K., de Weerd, S., Cikot, R. J., Steegers, E. A., & Braspenning, J. C. (2003). Validation of the dutch short form of the state scale of the Spielberger State-Trait Anxiety Inventory: considerations for usage in screening outcomes. Public Health Genomics, 6(2), 84-87.*

*Page 14*

15. Some people have already been vaccinated against meningococcal types A, C, W and Y. This is a different vaccination than the meningococcal vaccination that was offered to babies between 2002 and May 2018. That vaccination only protects against meningococci type C.

Have you already been vaccinated against meningococcal types A, C, W and Y?

⃝ Yes, for a travel abroad (info button: the vaccination is advised for travel to Saudi Arabia, Mongolia and parts of Africa)

⃝ Yes, following the news reports about meningococci earlier this year

⃝ Yes, but for a different reason…

⃝ No

⃝ I don't know

**Routing: Only if 'No' or 'I don't know' at 15.*

16. Do you want to be vaccinated against meningococcal type W?

Certainly not ⃝ ⃝ ⃝ ⃝ ⃝ ⃝ ⃝ Certainly yes

*Page 15*

**Routing: only if the respondent has (a) child(ren) under the age of 18 years*

17. Was one or more of your children born in 2001, 2002, 2003, 2004 or 2005?

⃝ Yes

⃝ No

**Routing: Only if yes at 17.*

18. Children born in 2001 to 2005 were invited in 2018 and 2019 for a catch-up vaccination against meningococcal types A, C, W and Y. Did your child(ren) receive the vaccination against meningococcal types A, C, W and Y?

⃝ Yes

⃝ No

⃝ I do not know

⃝ Other, namely…

*Page 16*

**Routing: only if the respondent has (a) child(ren) under the age of 18 years*

19. Has your youngest child already been vaccinated against meningococcal types A, C, W and Y. Note: This question may be the same for you as the previous question. We kindly ask you to fill in this question anyway.

⃝ Yes in 2018 or 2019 according to the National Immunization Program, when he/she was approximately 14 months old (your child was born in 2017 or 2018)

⃝ Yes in 2018 or 2019, after he/she was invited for the catch-up vaccination (your child was born in 2001, 2002, 2003, 2004 or 2005)

⃝ Yes, for a travel abroad (info button: the vaccination is advised for travel to Saudi Arabia, Mongolia and parts of Africa)

⃝ Yes, following the news reports about meningococci over the past few years

⃝ Yes, but for a different reason…

⃝ No

⃝ I do not know

*Page 17*

**Routing: only if the respondent has (a) child(ren) under the age of 18 years and 'No' or 'I don't know' for question 19.*

20. Do you want your youngest child to be vaccinated against meningococcal disease type A, C, W, and Y?

Certainly not ⃝ ⃝ ⃝ ⃝ ⃝ ⃝ ⃝ Certainly yes

*Page 18*

**Routing: only if the respondent has (a) child(ren) under the age of 18 years*

21. All children are offered vaccinations against infectious diseases through the National Immunization Program (NIP). Will your child receive the vaccinations according to this program?

⃝ Yes, my child has received all vaccinations that my child could receive for his/her age from the National Immunization Program

⃝ Yes, partly. My child started vaccinating later and/or did not receive all the vaccinations that my child could receive for his/her age from the National Immunization Program

⃝ No, my child does not participate in the National Immunization Program

⃝ I don't know

⃝ Other, namely…

*Page 19*

The following questions are about your trust in different authorities.

22. The Dutch government determines the policy for infectious diseases and vaccinations. What do you think about our government when it comes to infectious diseases and vaccinations? Indicate to what extent you agree or disagree with the statements.

|  | Completely disagree | Disagree | Do not agree nor disagree | Agree | Completely agree |
| --- | --- | --- | --- | --- | --- |
| The government has sufficient knowledge and skills with regard to infectious diseases and vaccinations. | ⃝ | ⃝ | ⃝ | ⃝ | ⃝ |
| The government communicates openly about infectious diseases and vaccinations. | ⃝ | ⃝ | ⃝ | ⃝ | ⃝ |
| The government puts the health of citizens above economic interests when it comes to vaccinations. | ⃝ | ⃝ | ⃝ | ⃝ | ⃝ |

*Page 20*

23. The National Institute for Public Health and the Environment (RIVM) keeps an eye on the risks to public health posed by infectious diseases. Furthermore, the RIVM implements the policy of the government regarding infectious diseases and vaccinations. What do you think about the RIVM when it comes to infectious diseases and vaccinations? Indicate to what extent you agree or disagree with the statements.

|  | Completely disagree | Disagree | Do not agree nor disagree | Agree | Completely agree |
| --- | --- | --- | --- | --- | --- |
| The RIVM has sufficient knowledge and skills with regard to infectious diseases and vaccinations. | ⃝ | ⃝ | ⃝ | ⃝ | ⃝ |
| The RIVM communicates openly about infectious diseases and vaccinations. | ⃝ | ⃝ | ⃝ | ⃝ | ⃝ |
| The RIVM puts the health of citizens above economic interests when it comes to vaccinations. | ⃝ | ⃝ | ⃝ | ⃝ | ⃝ |

*Page 21*

24. Vaccines against infectious diseases are made and sold by pharmaceutical companies. What do you think about pharmaceutical companies when it comes to vaccines? Indicate to what extent you agree or disagree with the statements.

|  | Completely disagree | Disagree | Do not agree nor disagree | Agree | Completely agree |
| --- | --- | --- | --- | --- | --- |
| Pharmaceutical companies have sufficient knowledge and skills with regard to infectious diseases and vaccinations. | ⃝ | ⃝ | ⃝ | ⃝ | ⃝ |
| Pharmaceutical companies communicate openly about infectious diseases and vaccinations. | ⃝ | ⃝ | ⃝ | ⃝ | ⃝ |
| Pharmaceutical companies put the health of citizens above economic interests when it comes to vaccinations. | ⃝ | ⃝ | ⃝ | ⃝ | ⃝ |

*Page 22*

This is the end of the questionnaire. Thank you for your participation! Would you like more information about meningococcal disease or about vaccinations? Visit the website of the RIVM or the website of the National Immunization Program.
