# Supplementary material for: Dynamics in public perceptions and media coverage during an ongoing outbreak of meningococcal W disease in the Netherlands
Source: BMC Public Health. 2022 Apr 1;22:633. doi: 10.1186/s12889-022-12920-8 (PMC8973985; doi:10.1186/s12889-022-12920-8)
Supplement: Supplementary file 3 — Additional file 3. Table S1. (Differences between respondents who participated in all three survey waves (S1-S3) and respondents who did not participate in all three survey waves. Differences were studied in demographic characteristics (age, sex and education level) and all outcome measures (shown in Table 1 main text) from the first survey (S1)). [file 12889_2022_12920_MOESM3_ESM.docx]

**Supplementary File 3**

*Supplementary File Table 1. Differences between respondents who participated in all three survey waves (S1-S3) and respondents who did not participate in all three survey waves. Differences were studied in demographic characteristics (age, sex and education level) and all outcome measures (shown in Table 1 main text) from the first survey (S1).*

|  | **Respondent group** | **Respondents all three waves** | | **Respondents not all three waves** | |  |
| --- | --- | --- | --- | --- | --- | --- |
| **Results independent**  **t-tests** | **Variable** | **N** | **Mean**  **(Std. Dev.)** | **N** | **Mean**  **(Std. Dev.)** | **P-value** |
|  | *Age in years* | 542 | 49.1 (13.6) | 527 | 45.8 (12.8) | 0.032 |
| **Results chi^2^ tests** | **Variable and answer categories** | **N** | **%** | **N** | **%** | **P-value** |
|  | Sex: |  |  |  |  | 0.983 |
|  | - *Male* | 265 | 48.9% | 258 | 49.0% |  |
|  | - *Female* | 277 | 51.1% | 269 | 51.0% |  |
|  | *Education level:* |  |  |  |  | 0.007 |
|  | - *Low* | 155 | 28.6% | 191 | 36.2% |  |
|  | - *Intermediate* | 232 | 42.8% | 181 | 34.3% |  |
|  | - *High* | 155 | 28.6% | 155 | 29.4% |  |
|  | *Subgroup:* |  |  |  |  | 0.005 |
|  | - *Parents (T)** | 111 | 20.5% | 102 | 19.4% |  |
|  | - *Parents (O)** | 174 | 32.1% | 218 | 41.4% |  |
|  | - *Individuals (NC)** | 257 | 47.4% | 207 | 39.3% |  |
|  | *Total* | 542 | 100% | 527 | 100% | - |

** Parents of teenagers invited for the menACWY catch-up vaccination campaign (parents (T)), parents of children under the age of 18 who were not invited for a menACWY vaccination (parents (O)), and individuals with no children under the age of 18 (individuals (NC)).*
