# Supplementary material for: Dynamics in public perceptions and media coverage during an ongoing outbreak of meningococcal W disease in the Netherlands
Source: BMC Public Health. 2022 Apr 1;22:633. doi: 10.1186/s12889-022-12920-8 (PMC8973985; doi:10.1186/s12889-022-12920-8)
Supplement: Supplementary file 4 — Additional file 4. Table S2. (Means (M) and standard deviations (SD) of perceptions, trust, and willingness to vaccinate among individuals (NC), parents (T + O), Parents (T) and parents (O). These descriptives include only the respondents who participated in all three survey rounds (T1-T3)). Table 3 (Multilevel analyses in Individuals (NC) and Parents (T + O). All analyses were controlled for age, sex and education level.). Table S4. (Multilevel analyses in Parents (T and O). All analyses were controlled for age, sex and education level.). [file 12889_2022_12920_MOESM4_ESM.docx]

**Supplementary File 4**

*Supplementary File Table 2. Means (M) and standard deviations (SD) of perceptions, trust, and willingness to vaccinate among individuals (NC), parents (T+O), Parents (T) and parents (O). These descriptives include only the respondents who participated in all three survey rounds (T1-T3).*

|  |  | Individuals (NC) | | | Parents (T+O) | | | Parents (T) | | | Parents (O) | | |
| --- | --- | --- | --- | --- | --- | --- | --- | --- | --- | --- | --- | --- | --- |
|  |  | N | M | SD | N | M | SD | N | M | SD | N | M | SD |
| Perceived probability IMD - self | T1 | 257 | 1.9 | 1.2 | 285 | 1.8 | 1.1 | 111 | 1.9 | 1.2 | 174 | 1.8 | 1.1 |
|  | T2 | 257 | 1.8 | 1.2 | 285 | 1.9 | 1.2 | 111 | 1.9 | 1.2 | 174 | 1.9 | 1.2 |
|  | T3 | 257 | 1.8 | 1.2 | 285 | 1.8 | 1.2 | 111 | 1.7 | 1.3 | 174 | 1.9 | 1.2 |
| Perceived probability IMD – child* | T1 |  |  |  | 285 | 2.2 | 1.1 | 111 | 2.1 | 1.1 | 174 | 2.3 | 1.2 |
|  | T2 |  |  |  | 285 | 2.2 | 1.1 | 111 | 2.1 | 1.0 | 174 | 2.3 | 1.1 |
|  | T3 |  |  |  | 285 | 2.0 | 1.3 | 111 | 1.7 | 1.3 | 174 | 2.2 | 1.3 |
| Perceived severity IMD – self | T1 | 257 | 4.7 | 1.3 | 285 | 4.6 | 1.2 | 111 | 4.7 | 1.2 | 174 | 4.6 | 1.3 |
|  | T2 | 257 | 5.2 | 1.1 | 285 | 5.0 | 1.2 | 111 | 5.2 | 1.0 | 174 | 4.8 | 1.3 |
|  | T3 | 257 | 5.0 | 1.1 | 285 | 5.0 | 1.1 | 111 | 5.2 | 1.1 | 174 | 4.8 | 1.2 |
| Perceived severity IMD – child* | T1 |  |  |  | 285 | 5.2 | 1.2 | 111 | 5.2 | 1.2 | 174 | 5.1 | 1.2 |
|  | T2 |  |  |  | 285 | 5.5 | 1.1 | 111 | 5.5 | 1.0 | 174 | 5.4 | 1.2 |
|  | T3 |  |  |  | 285 | 5.5 | 1.0 | 111 | 5.6 | 0.9 | 174 | 5.4 | 1.1 |
| Attitude menACWY vaccination | T1 | 257 | 4.9 | 1.2 | 285 | 4.8 | 1.3 | 111 | 5.0 | 1.1 | 174 | 4.7 | 1.3 |
|  | T2 | 257 | 5.2 | 1.1 | 285 | 5.1 | 1.2 | 111 | 5.3 | 1.1 | 174 | 5.1 | 1.2 |
|  | T3 | 257 | 5.4 | 0.8 | 285 | 5.3 | 1.1 | 111 | 5.5 | 1.0 | 174 | 5.3 | 1.1 |
| Trust government | T1 | 257 | 2.4 | 0.8 | 285 | 2.5 | 0.8 | 111 | 2.5 | 0.8 | 174 | 2.4 | 0.9 |
|  | T2 | 257 | 2.5 | 0.8 | 285 | 2.6 | 0.8 | 111 | 2.5 | 0.8 | 174 | 2.6 | 0.9 |
|  | T3 | 257 | 2.7 | 0.8 | 285 | 2.7 | 0.9 | 106 | 2.7 | 0.8 | 174 | 2.7 | 0.9 |
| Trust RIVM | T1 | 257 | 2.6 | 0.8 | 285 | 2.6 | 0.8 | 111 | 2.6 | 0.8 | 174 | 2.6 | 0.8 |
|  | T2 | 257 | 2.7 | 0.8 | 285 | 2.8 | 0.8 | 111 | 2.8 | 0.8 | 174 | 2.8 | 0.8 |
|  | T3 | 257 | 2.9 | 0.8 | 285 | 2.9 | 0.8 | 111 | 2.9 | 0.7 | 174 | 2.9 | 0.9 |
| Trust pharmaceutical companies | T1 | 257 | 1.8 | 0.7 | 285 | 1.9 | 0.7 | 111 | 2.0 | 0.6 | 174 | 1.9 | 0.8 |
|  | T2 | 257 | 1.9 | 0.7 | 285 | 2.1 | 0.8 | 111 | 2.1 | 0.7 | 174 | 2.1 | 0.8 |
|  | T3 | 257 | 2.0 | 0.7 | 285 | 2.2 | 0.8 | 111 | 2.3 | 0.7 | 174 | 2.1 | 0.9 |
| MenACWY willingness to vaccinate - self | T1 | 252 | 3.6 | 1.6 | 274 | 3.6 | 1.6 | 107 | 3.8 | 1.7 | 167 | 3.6 | 1.6 |
|  | T2 | 247 | 4.1 | 1.6 | 268 | 4.1 | 1.6 | 106** | 4.2 | 1.7 | 162 | 4.1 | 1.6 |
|  | T3 | 236 | 4.2 | 1.7 | 266 | 4.2 | 1.7 | 102 | 4.3 | 1.7 | 164 | 4.2 | 1.6 |
| MenACWY willingness to vaccinate - child* | T1 |  |  |  | 266 | 4.4 | 1.5 | 102 | 4.4 | 1.4 | 164 | 4.4 | 1.5 |
|  | T2 |  |  |  | 242 | 5.0 | 1.4 | 93 | 5.1 | 1.4 | 149 | 5.0 | 1.4 |
|  | T3 |  |  |  | 149 | 5.0 | 1.4 | 13*** | 4.6 | 1.4 | 136 | 5.0 | 1.4 |

** Only assessed among parents*

*** Only assessed among respondents who indicated that their child had not yet received the menACWY vaccination*

**** Due to a fault in the survey system, 72 parents have not received the questions regarding their trust in the government at T3. Three days after the T3 data collection, these respondents were asked to fill in these questions in retrospect. The data was checked for irregularities and in was decided to include the 67/72 respondents who responded to the late request for the trust questions.*

*Supplementary File Table 3. Multilevel analyses in Individuals (NC) and Parents (T+O). All analyses were controlled for age, sex and education level.*

|  | *Independent variables** | *Model 1* |  |  |  | *Model 2* |  |  |  |
| --- | --- | --- | --- | --- | --- | --- | --- | --- | --- |
|  |  | *B* | *P* | *95% CI* |  | *B* | *P* | *95% CI* |  |
| *Perceived*  probability IMD *- self* | T1 | 0.0 | 0.417 | -0.1 | 0.1 | 0.1 | 0.065 | 0.0 | 0.2 |
|  | T3 | 0.0 | 0.576 | -0.1 | 0.1 | 0.0 | 0.585 | -0.1 | 0.2 |
|  | Parents | 0.0 | 0.567 | -0.2 | 0.1 | 0.1 | 0.441 | -0.1 | 0.2 |
|  | T1*parents |  |  |  |  | -0.2 | 0.078 | -0.3 | 0.0 |
|  | T3*parents |  |  |  |  | -0.1 | 0.206 | -0.3 | 0.1 |
| *Perceived*  probability IMD - *child*** | T1 | -0.1 | 0.031 | -0.3 | 0.0 |  |  |  |  |
|  | T3 | -0.4 | 0.000 | -0.5 | -0.2 |  |  |  |  |
| *Perceived severity IMD - self* | T1 | -0.4 | 0.000 | -0.5 | -0.3 | -0.4 | 0.000 | -0.5 | -0.3 |
|  | T3 | -0.1 | 0.145 | -0.2 | 0.0 | -0.1 | 0.064 | -0.3 | 0.0 |
|  | Parents | -0.1 | 0.250 | -0.2 | 0.1 | -0.1 | 0.180 | -0.3 | 0.1 |
|  | T1*parents |  |  |  |  | 0.0 | 0.702 | -0.1 | 0.2 |
|  | T3*parents |  |  |  |  | 0.1 | 0.239 | -0.1 | 0.3 |
| *Perceived severity IMD – child*** | T1 | -0.3 | 0.000 | -0.4 | -0.2 |  |  |  |  |
|  | T3 | 0.0 | 0.913 | -0.1 | 0.1 |  |  |  |  |
| *Attitude menACWY vaccination* | T1 | -0.3 | 0.000 | -0.4 | -0.2 | -0.3 | 0.000 | -0.4 | -0.2 |
|  | T3 | 0.2 | 0.000 | 0.1 | 0.3 | 0.2 | 0.000 | 0.1 | 0.3 |
|  | Parents | -0.1 | 0.331 | -0.2 | 0.1 | -0.1 | 0.309 | -0.3 | 0.1 |
|  | T1*parents |  |  |  |  | 0.0 | 0.768 | -0.1 | 0.2 |
|  | T3*parents |  |  |  |  | 0.0 | 0.670 | -0.1 | 0.2 |
| *Trust RIVM* | T1 | -0.1 | 0.000 | -0.2 | -0.1 | -0.1 | 0.004 | -0.2 | 0.0 |
|  | T3 | 0.1 | 0.000 | 0.1 | 0.2 | 0.1 | 0.001 | 0.1 | 0.2 |
|  | Parents | -0.1 | 0.186 | -0.2 | 0.0 | 0.0 | 0.407 | -0.2 | 0.1 |
|  | T1*parents |  |  |  |  | 0.0 | 0.659 | -0.1 | 0.1 |
|  | T3*parents |  |  |  |  | 0.0 | 0.780 | -0.1 | 0.1 |
| *Trust government* | T1 | -0.1 | 0.000 | -0.2 | 0.0 | -0.1 | 0.031 | -0.2 | 0.0 |
|  | T3 | 0.2 | 0.000 | 0.1 | 0.2 | 0.2 | 0.000 | 0.1 | 0.3 |
|  | Parents | 0.0 | 0.589 | -0.1 | 0.1 | 0.0 | 0.979 | -0.1 | 0.1 |
|  | T1*parents |  |  |  |  | 0.0 | 0.625 | -0.1 | 0.1 |
|  | T3*parents |  |  |  |  | -0.1 | 0.240 | -0.2 | 0.1 |
| *Trust pharmaceutical companies* | T1 | -0.1 | 0.000 | -0.2 | -0.1 | -0.1 | 0.009 | -0.2 | 0.0 |
|  | T3 | 0.1 | 0.001 | 0.0 | 0.2 | 0.1 | 0.029 | 0.0 | 0.2 |
|  | Parents | 0.0 | 0.384 | 0.0 | 0.1 | 0.0 | 0.404 | -0.1 | 0.2 |
|  | T1*parents |  |  |  |  | 0.0 | 0.698 | -0.1 | 0.1 |
|  | T3*parents |  |  |  |  | 0.0 | 0.792 | -0.1 | 0.1 |
| *MenACWY willingness to vaccinate - self* | T1 | -0.5 | 0.000 | -0.6 | -0.4 | -0.5 | 0.000 | -0.7 | -0.4 |
|  | T3 | 0.1 | 0.041 | 0.0 | 0.3 | 0.1 | 0.364 | -0.1 | 0.3 |
|  | Parents | -0.1 | 0.163 | -0.3 | 0.1 | -0.2 | 0.130 | -0.4 | 0.1 |
|  | T1*parents |  |  |  |  | 0.1 | 0.587 | -0.2 | 0.3 |
|  | T3*parents |  |  |  |  | 0.1 | 0.497 | -0.2 | 0.3 |
| *MenACWY willingness to vaccinate – child*** | T1 | -0.6 | 0.000 | -0.7 | -0.5 |  |  |  |  |
|  | T3 | 0.1 | 0.537 | -0.1 | 0.2 |  |  |  |  |

** Reference groups: T2 and individuals (NC)*

*** Only assessed in parents (T and O)*

*Supplementary File Table 4. Multilevel analyses in Parents (T and O). All analyses were controlled for age, sex and education level.*

|  |  | *Model 1* |  |  |  | *Model 2* |  |  |  |
| --- | --- | --- | --- | --- | --- | --- | --- | --- | --- |
| *Dependent variable* | *Independent variable** | *B* | *P-value* | *95% CI* |  | *B* | *P-value* | *95% CI* |  |
| *Perceived probability IMD - child* | *T1* | -0.1 | 0.030 | -0.3 | 0.0 | -0,1 | 0,331 | -0,2 | 0,1 |
|  | *T3* | -0.4 | 0.000 | -0.5 | -0.2 | -0,1 | 0,150 | -0,3 | 0,1 |
|  | *Parents (T)* | -0.1 | 0.368 | -0.3 | 0.1 | 0,1 | 0,361 | -0,1 | 0,4 |
|  | *T1*parents (T)* |  |  |  |  | -0,2 | 0,204 | -0,4 | 0,1 |
|  | *T3*parents (T)* |  |  |  |  | -0,6 | 0,000 | -0,9 | -0,3 |
| *Perceived probability IMD – self* | *T1* | 0,0 | 0,558 | -0,2 | 0,1 | 0,0 | 0,522 | -0,2 | 0,1 |
|  | *T3* | -0,1 | 0,233 | -0,2 | 0,1 | 0,0 | 0,768 | -0,2 | 0,2 |
|  | *Parents (T)* | 0,0 | 0,694 | -0,1 | 0,2 | 0,0 | 0,701 | -0,2 | 0,3 |
|  | *T1*parents (T)* |  |  |  |  | 0,0 | 0,769 | -0,2 | 0,3 |
|  | *T3*parents (T)* |  |  |  |  | -0,2 | 0,308 | -0,5 | 0,1 |
| *Perceived severity IMD - child* | *T1* | -0.3 | 0.000 | -0.4 | -0.2 | -0,3 | 0,000 | -0,4 | -0,1 |
|  | *T3* | 0.0 | 0.929 | -0.1 | 0.1 | 0,0 | 0,985 | -0,2 | 0,2 |
|  | *Parents (T)* | 0.2 | 0.061 | 0.0 | 0.4 | 0,2 | 0,138 | -0,1 | 0,4 |
|  | *T1*parents (T)* |  |  |  |  | 0,0 | 0,894 | -0,2 | 0,2 |
|  | *T3*parents (T)* |  |  |  |  | 0,0 | 0,890 | -0,3 | 0,3 |
| *Perceived severity IMD - self* | *T1* | -0,3 | 0,000 | -0,5 | -0,2 | -0,3 | 0,000 | -0,4 | -0,1 |
|  | *T3* | 0,0 | 0,768 | -0,2 | 0,1 | 0,0 | 0,975 | -0,2 | 0,2 |
|  | *Parents (T)* | 0,3 | 0,003 | 0,1 | 0,5 | 0,4 | 0,002 | 0,1 | 0,6 |
|  | *T1*parents (T)* |  |  |  |  | -0,2 | 0,123 | -0,4 | 0,1 |
|  | *T3*parents (T)* |  |  |  |  | -0,1 | 0,719 | -0,3 | 0,2 |
| *Attitude menACWY vaccination* | *T1* | -0,3 | 0,000 | -0,4 | -0,2 | -0,3 | 0,000 | -0,4 | -0,1 |
|  | *T3* | 0,2 | 0,000 | 0,1 | 0,4 | 0,2 | 0,000 | 0,1 | 0,4 |
|  | *Parents (T)* | 0,2 | 0,097 | 0,0 | 0,4 | 0,2 | 0,085 | 0,0 | 0,5 |
|  | *T1*parents (T)* |  |  |  |  | -0,1 | 0,514 | -0,3 | 0,1 |
|  | *T3*parents (T)* |  |  |  |  | 0,0 | 0,863 | -0,2 | 0,2 |
| *Trust RIVM* | *T1* | -0,1 | 0,000 | -0,2 | -0,1 | -0,1 | 0,002 | -0,2 | 0,0 |
|  | *T3* | 0,1 | 0,001 | 0,0 | 0,2 | 0,1 | 0,025 | 0,0 | 0,2 |
|  | *Parents (T)* | 0,1 | 0,213 | -0,1 | 0,2 | 0,1 | 0,336 | -0,1 | 0,2 |
|  | *T1*parents (T)* |  |  |  |  | 0,0 | 0,990 | -0,1 | 0,1 |
|  | *T3*parents(T)* |  |  |  |  | 0,0 | 0,642 | -0,1 | 0,2 |
| *Trust government* | *T1* | -0,1 | 0,002 | -0,2 | 0,0 | -0,2 | 0,000 | -0,3 | -0,1 |
|  | *T3* | 0,1 | 0,001 | 0,1 | 0,2 | 0,1 | 0,121 | 0,0 | 0,2 |
|  | *Parents (T)* | 0,1 | 0,074 | 0,0 | 0,3 | 0,0 | 0,783 | -0,1 | 0,2 |
|  | *T1*parents(T)* |  |  |  |  | 0,1 | 0,060 | 0,0 | 0,3 |
|  | *T3*parents(T)* |  |  |  |  | 0,2 | 0,066 | 0,0 | 0,3 |
| *Trust pharmaceutical companies* | *T1* | -0,1 | 0,001 | -0,2 | 0,0 | -0,1 | 0,001 | -0,2 | -0,1 |
|  | *T3* | 0,1 | 0,007 | 0,0 | 0,2 | 0,0 | 0,369 | -0,1 | 0,1 |
|  | *Parents (T)* | 0,1 | 0,036 | 0,0 | 0,3 | 0,1 | 0,448 | -0,1 | 0,2 |
|  | *T1*parents(T)* |  |  |  |  | 0,1 | 0,236 | -0,1 | 0,2 |
|  | *T3*parents(T)* |  |  |  |  | 0,2 | 0,051 | 0,0 | 0,3 |
| *MenACWY willingness to vaccinate - child* | *T1* | -0,6 | 0,000 | -0,7 | -0,5 | -0,5 | 0,000 | -0,6 | -0,3 |
|  | *T3* | 0,1 | 0,515 | -0,1 | 0,3 | 0,2 | 0,100 | 0,0 | 0,4 |
|  | *Parents (T)* | 0,1 | 0,631 | -0,2 | 0,3 | 0,3 | 0,049 | 0,0 | 0,7 |
|  | *T1*parents (T)* |  |  |  |  | -0,4 | 0,007 | -0,6 | -0,1 |
|  | *T3*parents (T)* |  |  |  |  | -0,6 | 0,064 | -1,2 | 0,0 |
| *MenACWY willingness to vaccinate - self* | *T1* | -0.5 | 0.000 | -0.6 | -0.3 | -0.5 | 0.000 | -0.7 | -0.3 |
|  | *T3* | 0.2 | 0.047 | 0.0 | 0.3 | 0.2 | 0.127 | 0.0 | 0.4 |
|  | *Parents (T)* | 0.1 | 0.388 | -0.2 | 0.4 | 0.1 | 0.489 | -0.2 | 0.5 |
|  | *T1*parents (T)* |  |  |  |  | 0.0 | 0.980 | -0.3 | 0.3 |
|  | *T3*parents (T)* |  |  |  |  | 0.0 | 0.958 | -0.3 | 0.4 |

***** *Reference groups: T2 and Parents (O)*
