# Supplementary material for: Dynamics in public perceptions and media coverage during an ongoing outbreak of meningococcal W disease in the Netherlands
Source: BMC Public Health. 2022 Apr 1;22:633. doi: 10.1186/s12889-022-12920-8 (PMC8973985; doi:10.1186/s12889-022-12920-8)
Supplement: Supplementary file 5 — Additional file 5 Table S5. (Number of newspaper articles about meningococcal W disease and/or the menACWY vaccination per survey period.). [file 12889_2022_12920_MOESM5_ESM.docx]

**Supplementary File 5**

*Supplementary File Table 5. Number of newspaper articles about meningococcal W disease and/or the menACWY vaccination per survey period.*

| *Survey period* | *Corresponding dates* | *Number of newspaper articles published* |
| --- | --- | --- |
| <S1 | 01/09/2017 – 12/12/2017 | 6 |
| S1-S2 | 13/12/2017 – 20/09/2018 | 41 |
| S2-S3 | 21/09/2018 – 14/07/2019 | 55 |
| >S3 | 15/07/2019 – 31/08/2019 | 1 |
| Total | 01/09/2017 – 31/08/2019 | 103 |
